# Supplementary material for: Discovery of an Endonuclease G-inhibitory Ku80-peptide protecting against leukemogenic rearrangements at the MLL breakpoint cluster
Source: Nat Commun. 2026 Apr 17;17:3562. doi: 10.1038/s41467-026-72034-2 (PMC13086865; doi:10.1038/s41467-026-72034-2)
Supplement: Supplementary file 1 — Supplementary Information [file 41467_2026_72034_MOESM1_ESM.pdf]

SUPPLEMENTARY INFORMATION

For manuscript entitled:

Discovery of an Endonuclease G-inhibitory Ku80-peptide protecting against leukemogenic rearrangements at the MLL breakpoint cluster

By:
Julia Eberle#, Ahmed Salem#, Mara Hofmann#, Anja Reisser#, Yasser B. Ruiz-Blanco#, Yasser Almeida-Hernandez, Boris Gole, Melanie Rall-Scharpf, Jessica Angulo-Capel, Thomas Monecke,
Elsa Sanchez-Garcia*, J. Christof M. Gebhardt*, and Lisa Wiesmüller*.

# These authors contributed equally: Julia Eberle, Ahmed Salem, Mara Hofmann, Anja Reisser, Yasser B. Ruiz-Blanco

- Corresponding authors

- **Supplementary Figures 1-13**
- **Supplementary Tables 1-3**
- **Supplementary Methods**
- **Supplementary References**

Supplementary Figures

Supplementary Figure 1.

EndoG-Ku80 complex formation and no association of anti-recombinative effect of Ku80-Ct on cell cycle changes or cell death.

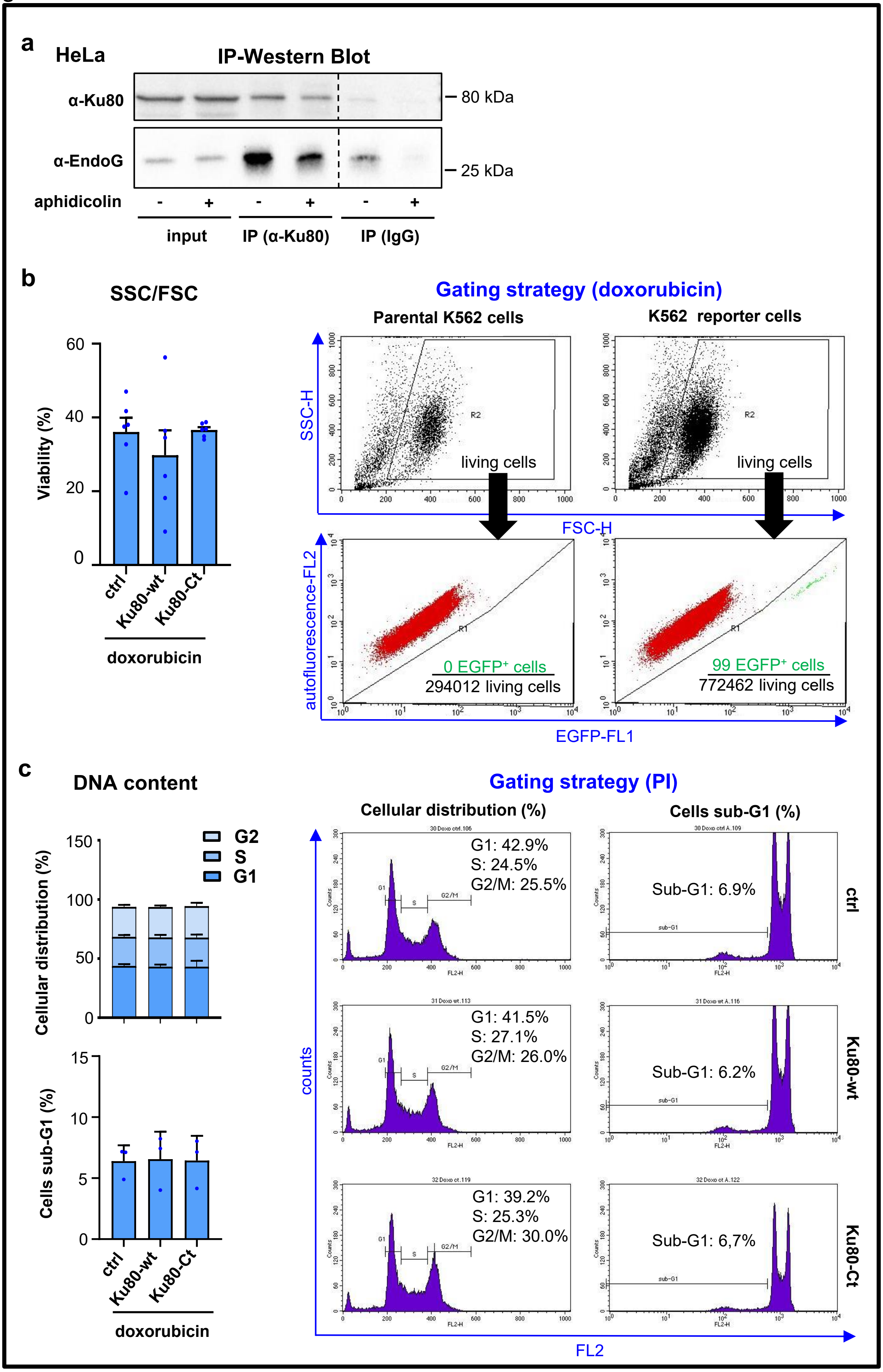

continued Supplementary Figure 1.

EndoG-Ku80 complex formation and no impact of anti-recombinative effect of Ku80-Ct on cell cycle changes or cell death.

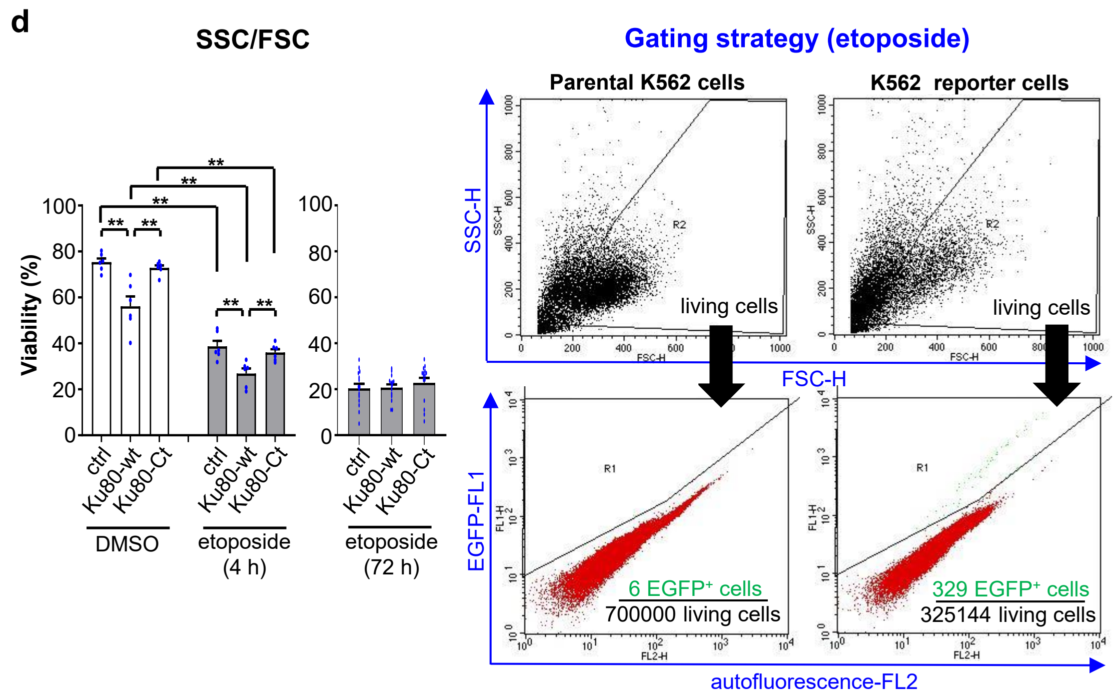

Supplementary Figure 1. EndoG-Ku80 complex formation and no impact of anti-recombinative effect of Ku80-Ct on cell cycle changes or cell death. (**a**) Immunoprecipitation. Pull-downs in HeLa cells (with or without treatment with 10 µM aphidicolin for 4 h) engaged polyclonal rabbit antibody directed against Ku80 (Santa Cruz Biotechnology, Dallas, Texas, USA, H-300, sc-9034) or control rabbit IgG (Santa Cruz, sc-2027). Subsequent Western blotting relied on the following primary antibodies: rabbit polyclonal anti-Ku80 (Santa Cruz Biotechnology, H-300, sc-9034) and mouse anti-EndoG (Santa Cruz, sc-365359). Peroxidase-coupled secondary antibodies were: goat anti-rabbit (Rockland, Pennsylvania, USA) and goat anti-mouse Fcγ (Jackson ImmunoResearch, Newmarket, UK), respectively. The panel shows a representative Western Blot of 3 independent experiments. IP = Immunoprecipitation. Stippled line implies cropping at that site. Uncropped Western blots in Source Data (uncropped images). (**b**) SSC/FSC-gating post doxorubicin treatment. Viabilities of K562 reporter cells ectopically expressing Ku80 variants post doxorubicin treatment (2 µM, 4 h followed by 72 h release) in Figure 1f were assessed by SSC/FSC-gating during FACS analysis. Data (n=6 samples from 3 independent experiments) are presented as mean +SEM. No statistically significant differences were found. Right: gating strategy illustrated on flow cytometry data of doxorubicin-treated parental and K562 reporter cells. Percentages of EGFP-positive cells in the living cell population (SSC-H/FSC-H gate) were calculated. Note that doxorubicin treatment reduces viability as described before[1]. Further note that for recombination measurements of doxorubicin-treated cells a specific autofluorescence-FL2/EGFP-FL1 gate was used due to the red autofluorescence of doxorubicin. (**c**) DNA content analysis. K562 reporter cells were treated with 2 µM doxorubicin and DNA content analysis performed following propidium iodide (PI) staining. No significant changes were detected in the percentages of G1-, S- or G2-phase cells as well as apoptotic cells indicated by a sub-G1 DNA content. Data from 3 independent experiments are presented as mean +SD. (**d**) SSC/FSC analysis and gating strategy post mock- and etoposide treatment. Viabilities of K562 reporter cells ectopically expressing Ku80 variants post DMSO or etoposide treatment (10 µM for 4 h followed by 72 h release or 10 µM for 72 h) in Figure 1g. Data are presented as mean +SEM (DMSO and etoposide, 4 h: n=6 samples from 3 independent experiments; etoposide, 72 h: n=15 samples from 5 independent experiments). Significances were calculated by Kruskal-Wallis H-test followed by two-tailed Mann-Whitney-U test (**$p<0.01$; ctrl, DMSO vs. etoposide 4 h: $p=0.0022$; Ku80-wt, DMSO vs. etoposide 4 h: $p=0.0022$; Ku80-Ct, DMSO vs. etoposide 4 h: $p=0.0022$; DMSO, ctrl vs. Ku80-wt: $p=0.0043$; DMSO, Ku80-wt vs. Ku80-Ct: $p=0.0043$; etoposide 4 h, ctrl vs. Ku80-wt: $p=0.0043$; etoposide 4 h, Ku80-wt vs. Ku80-Ct: $p=0.0087$). Gating strategy is illustrated on parental and reporter cells (Ku80-Ct positive) treated with etoposide for 72 h. Source data are provided as a Source Data file.

Supplementary Figure 2.

Ku80-Ct does not affect DSB repair.

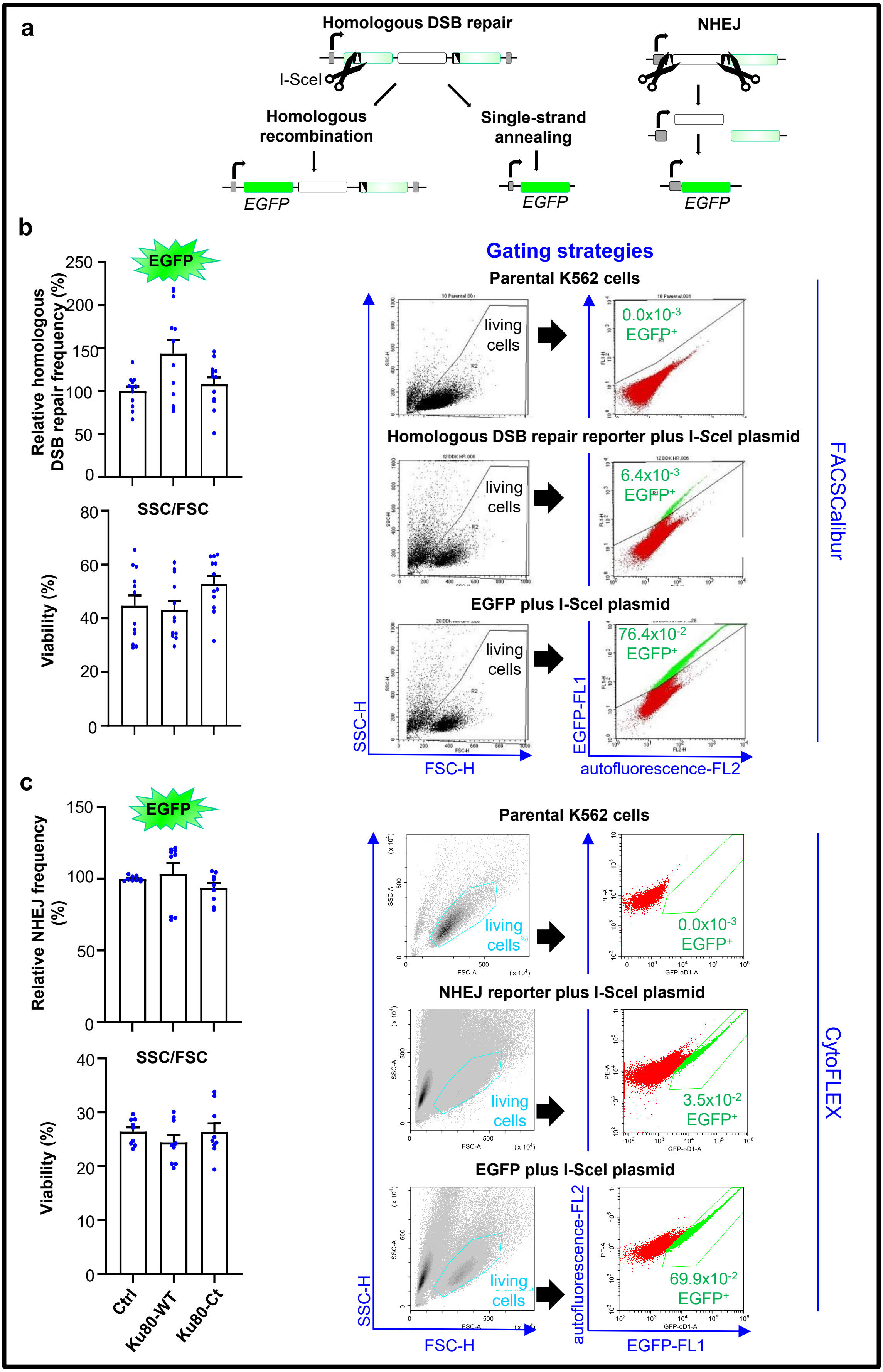

continued Supplementary Figure 2.
Ku80-Ct does not affect DSB repair.

Supplementary Figure 2. Ku80-Ct does not affect DSB repair. (**a**) Principles of homologous DSB repair and NHEJ assays. Repair measurements rely on quantification of EGFP-positivities following **I**-Sce**I**-mediated cleavage of reporter construct for homologous DSB repair between differently mutated *EGFP* genes encompassing a hygromycin resistance cassette as spacer sequence[2]. Both homologous recombination and single-strand annealing can reconstitute wild-type *EGFP*. NHEJ is triggered by cleavage at two **I**-Sce**I** sites and deletes the spacer, thereby positioning the *EGFP* coding sequence immediately downstream of the promoter[3]. (**b**) Homologous DSB repair analysis. Untreated K562 cells were electroporated with homologous DSB repair reporter plasmid (10 µg) as well as expression plasmids for the meganuclease **I**-Sce**I** (10 µg) and the different Ku80 variants (40 µg) and cultivated for 48 h. Left panels: Frequencies of EGFP-positive living cells in the population were normalized to the mean of ctrl values set to 100% per experiment (average: $3x10^{-3}$). Viabilities according to SSC/FSC-gating are displayed below. Data (n=12 samples from 4 independent experiments) are presented as mean +SEM. Right panels: Gating strategies illustrated on flow cytometry data obtained on a FACSCalibur™ (BD Biosciences, San Jose, California, USA) of parental K562 cells or K562 cells electroporated with homologous DSB repair reporter plus **I**-Sce**I** plasmid as well as EGFP and **I**-Sce**I** expression plasmids are shown. (**c**) NHEJ analysis. K562 cells were electroporated with NHEJ reporter (10 µg) as well as **I**-Sce**I** expression plasmid (10 µg) and the different Ku80 variants (40 µg) and cultivated for 48 h. Left panels: Frequencies of EGFP-positive living cells in the population, normalized to the mean of ctrl values (average: $4.4x10^{-2}$), and viabilities according to SSC/FSC-gating are shown. Data (n=9 samples from 3 independent experiments) are presented as mean +SEM. Right panels: Gating strategies on a CytoFLEX B3-R1-V0 flow cytometer with APD detectors and GFP-oD1 bandpass filter (Beckmann Coulter) of parental K562 cells, K562 cells electroporated with NHEJ reporter and **I**-Sce**I** plasmid as well as EGFP and **I**-Sce**I** expression plasmids are shown. No statistically significant differences were found by Kruskal-Wallis H-test followed by two-tailed Mann-Whitney-U test. Source data are provided as a Source Data file.

Supplementary Figure 3.

Effect of Ku80-Ct on Ku80 or γH2AX signals in the nucleus, cell cycle distribution and cell death.

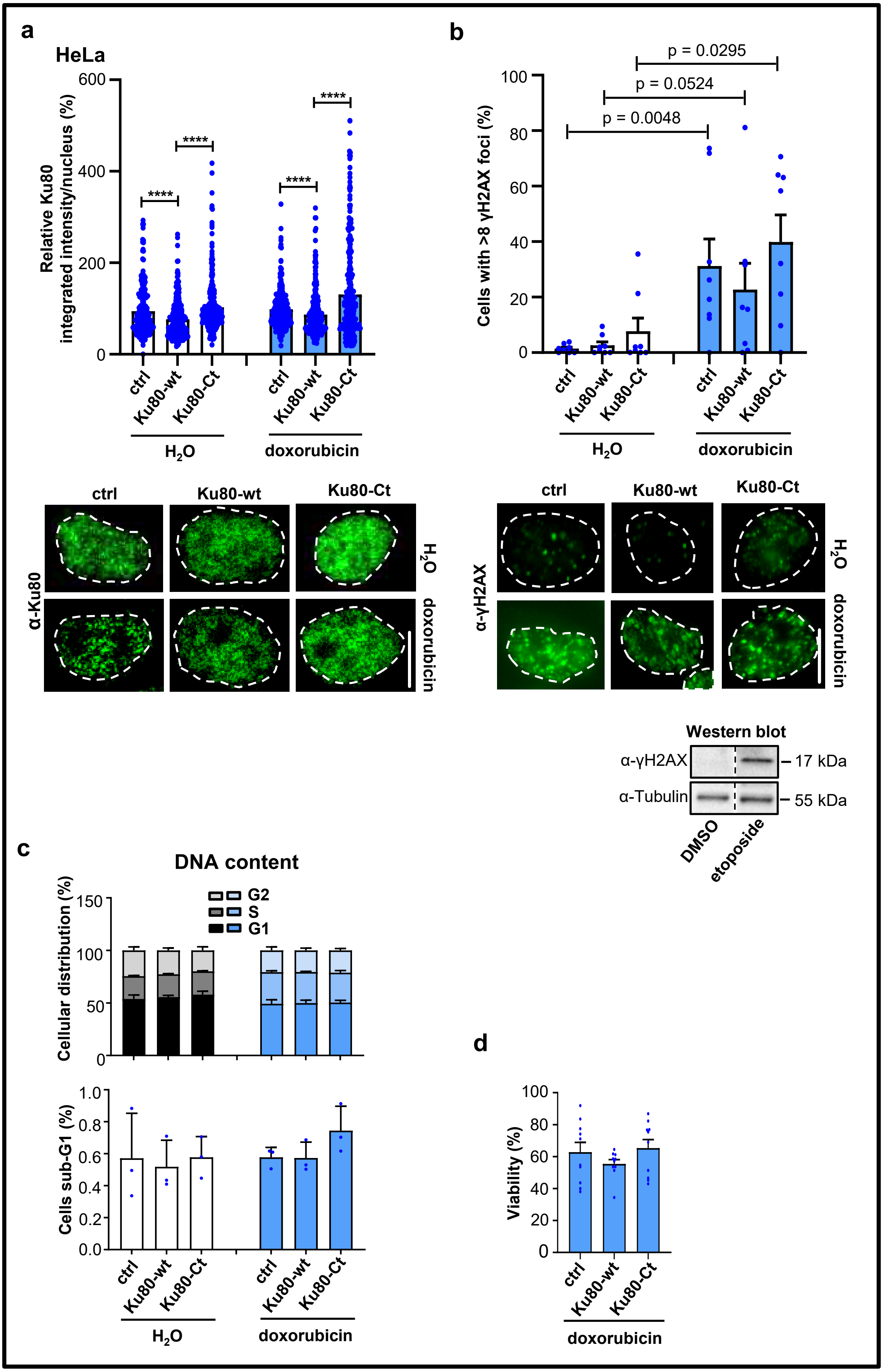

continued Supplementary Figure 3.

Effect of Ku80-Ct on Ku80 or γH2AX signals in the nucleus, cell cycle distribution and cell death.

Supplementary Figure 3. Effect of Ku80-Ct on Ku80 or γH2AX signals in the nucleus, cell cycle distribution and cell death. HeLa cells were transfected with expression constructs for Ku80-wt, Ku80-Ct or empty vector (ctrl), cultured and treated as in Figure 2b. Significances were calculated by Kruskal-Wallis H-test followed by two-tailed Mann-Whitney-U test. Scale bars indicate 10 µm. (**a**) Ku80 signals. Integrated nuclear Ku80 intensities were determined by immunofluorescence microscopy after H_2O or doxorubicin treatment and normalized to the mean ctrl values of doxorubicin-treated cells each. Mean values +SEM from n=228-313 nuclei of 3 independent experiments are shown (H_2O, ctrl: n=228; H_2O, Ku80-wt: n=290; H_2O, Ku80-Ct n=313; doxorubicin, ctrl: n=273; doxorubicin, Ku80-wt: n=288; doxorubicin, Ku80-Ct n=291; ****p<0.0001). (**b**) Nuclear DNA damage. γH2AX damage staining in HeLa cells (n=8 samples from 4 independent experiments) was evaluated to present cells with ≥9 foci per nucleus, showing an increase of damage in doxorubicin versus solvent (H_2O) treated cells. p-values<0.1 are indicated. Validation of anti-γH2AX Ser139 (Mouse, monoclonal, Clone JBW 301, Merck Millipore) was performed by Western blot analysis of γH2AX and α-Tubulin 4 h after DMSO/etoposide treatment. Representative blot from 2 independent experiments is shown. Stippled lines indicate cropping. Uncropped Western blot in Source Data (uncropped images). (**c**) DNA content analysis. Data from 3 independent experiments are presented as mean +SD. No significant changes were detected in the percentages of G1-, S- or G2-phase cells as well as apoptotic sub-G1 cells with H_2O or doxorubicin treatment. (**d**) Viabilities of HeLa cells ectopically expressing Ku80 variants for 24 h and subsequent treatment with doxorubicin. MTT assay after 24 h of treatment with 0.5 µM doxorubicin. Data (n=10 samples from 2 independent experiments) are presented as mean +SEM. No significant changes were detected. Source data are provided as a Source Data file.

Supplementary Figure 4.
Effects of Ku80 Ct-derived peptides on *MLL*bcr rearrangements and viability.

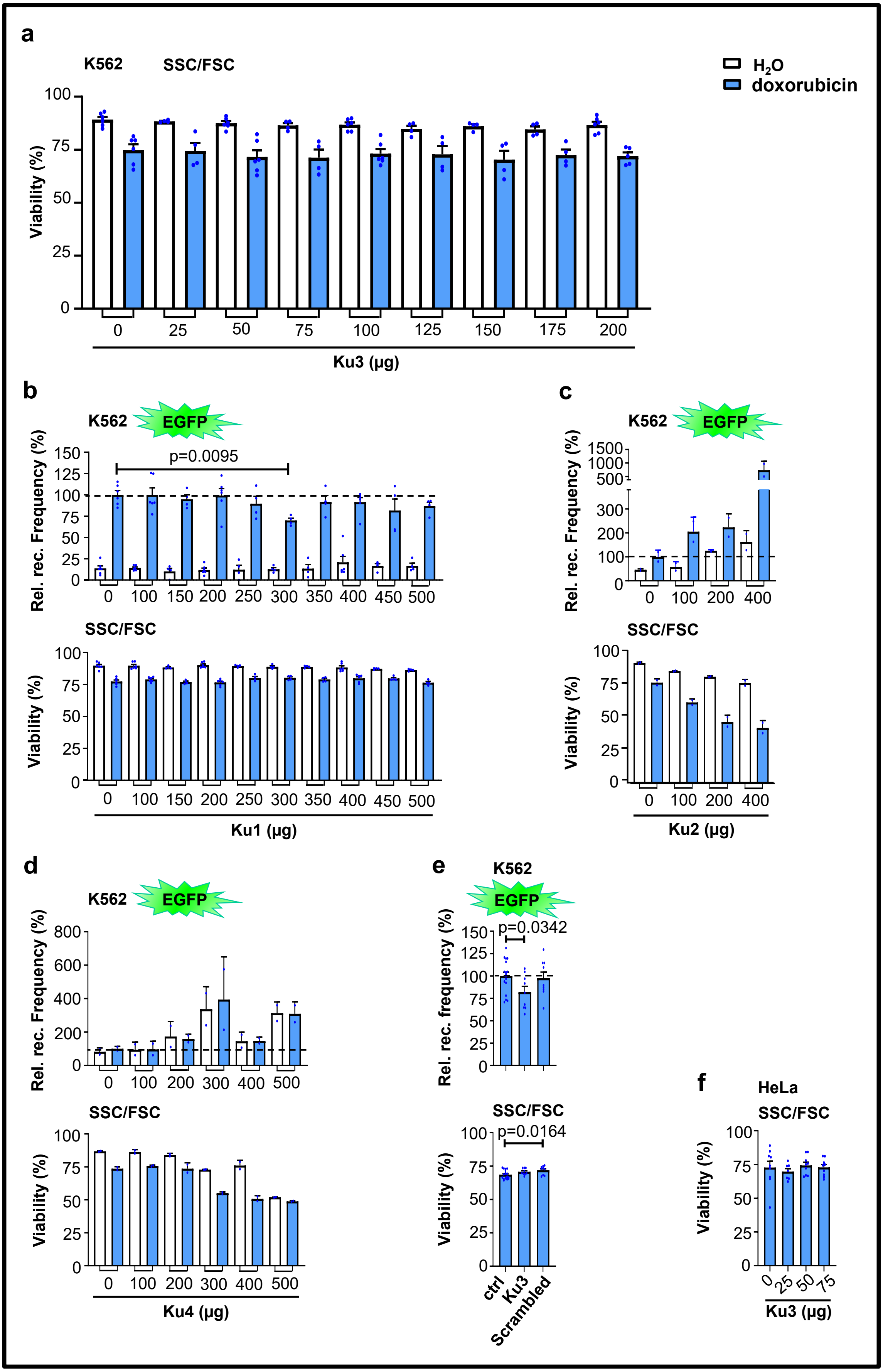

continued Supplementary Figure 4.

Effects of Ku80-Ct-derived peptides on *MLL*bcr rearrangements and viability.

Supplementary Figure 4. Effects of Ku80-Ct-derived peptides on *MLL*bcr rearrangements and viability. After nucleofection with increasing amounts of peptide, cells were cultured, H_2O (white bars) or doxorubicin (blue bars) treated, relative (rel.) recombination (rec.) frequencies determined and normalized to doxorubicin-treated cells as in Figure 4b and c, respectively. For assessment of viabilities SSC/FSC-gating was performed during each recombination experiment. Statistically significant differences were calculated for mock- and doxorubicin-treated cells separately by Kruskal-Wallis H-test ($p<0.1$) followed by two-tailed Mann-Whitney-U test and $p<0.05$ indicated. (**a**) Viabilities of K562 reporter cells. SSC/FSC-gating data were obtained during FACS analysis in Figure 4b and presented as mean +SEM. H_2O/doxorubicin, 0: n=6/6; 25: n=4/3; 50: n=6/6; 75: n=4/4; 100: n=6/6; 125: n=4/4; 150: n=4/4; 175: n=4/4; 200: n=6/6 samples from 2 (25, 75, 125, 150, 175) to 3 (0, 50, 100, 200) independent experiments. (**b**) Evaluation of K562 reporter cells after nucleofection with increasing amounts of Ku1 peptide. Data are presented as mean +SEM from 2 (150, 250, 300, 350, 450, 500) to 3 (0, 100, 200, 400) independent experiments with twice the number of samples each. Upper panel: Recombination measurements. Lower panel: SSC/FSC-gating. (**c**) Evaluation of K562 reporter cells after nucleofection with increasing amounts of Ku2 peptide. Data (n=2 samples) are presented as mean +SD. Upper panel: Recombination measurements. Lower panel: SSC/FSC-gating. (**d**) Evaluation of K562 reporter cells after nucleofection with increasing amounts of Ku4 peptide. Data (n=2 samples) are presented as mean +SD. Upper panel: Recombination measurements. Lower panel: SSC/FSC-gating. (**e**) Ku3 but not its scrambled version reduces *MLL*bcr rearrangements. K562 reporter cells after nucleofection with or without 100 µg of Ku3 or Scrambled. Data (ctrl: n=18, Ku3/Scrambled: n=9 samples from 3 independent experiments) are presented as mean +SEM. Upper panel: Recombination measurements. Lower panel: SSC/FSC-gating. (**f**) Viabilities of HeLa reporter cells. SSC/FSC-gating data were obtained during FACS analysis in Figure 4c and are presented as mean +SEM; 0: n=9; 25: n=7; 50: n=9; 75: n=9 samples from 4 (25) to 5 (0, 50, 75) independent experiments. Source data are provided as a Source Data file.

Supplementary Figure 5.
Ku3 interferes with *MLL*bcr breakage independently of DNA-PK activity.

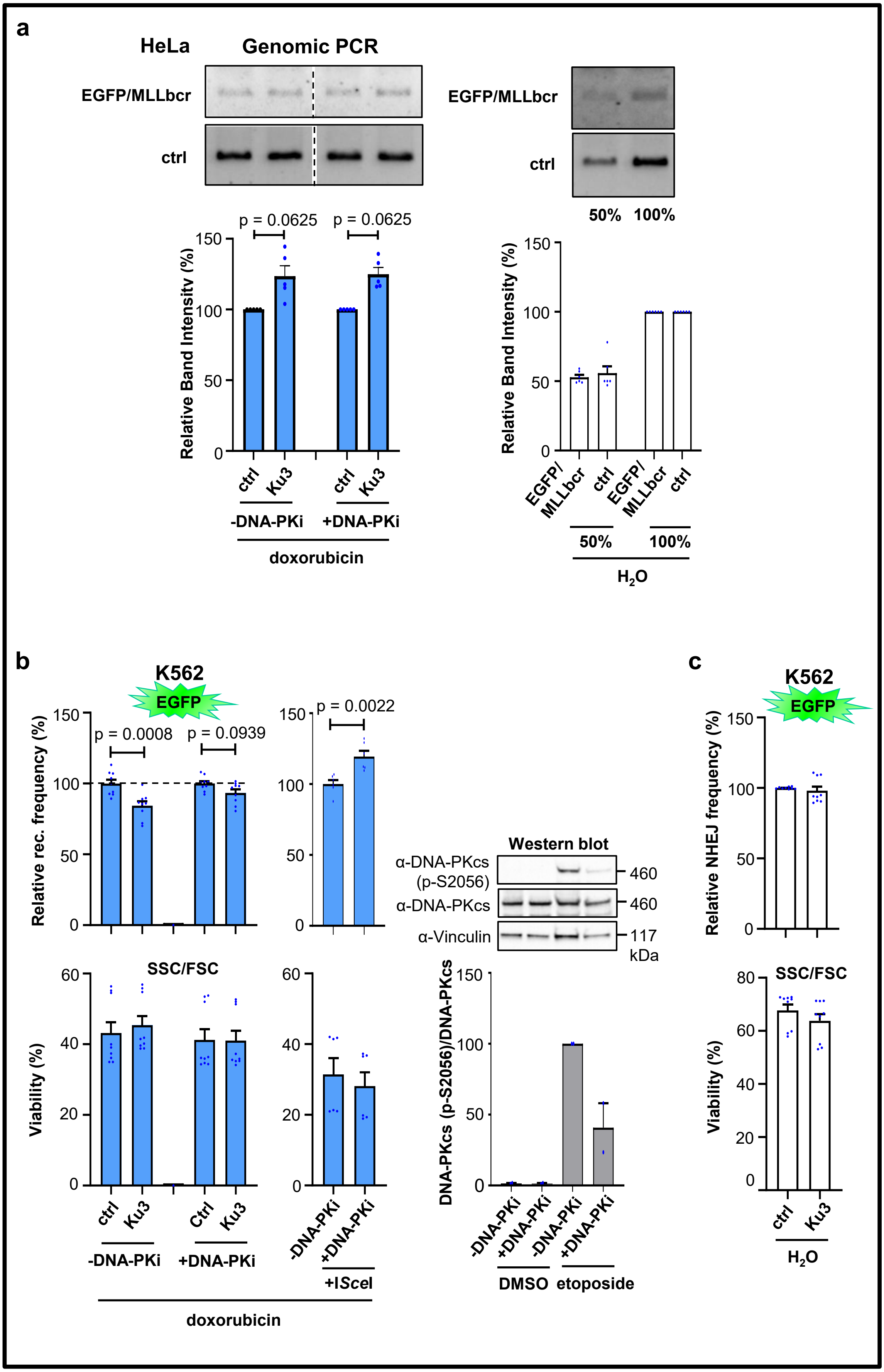

continued Supplementary Figure 5.
Ku3 interferes with *MLL*bcr breakage independently of DNA-PK activity.

Supplementary Figure 5. Ku3 interferes with *MLL*bcr breakage independently of DNA-PK activity. (**a**) Genomic PCR in HeLa cells. Genomic PCR analysis was performed on HeLa reporter cells nucleofected with or without 100 µg Ku3 and treated with doxorubicin (0.5 µM, 4 h). Cells were additionally treated with or without 1 µM DNA-PK inhibitor NU7441 (DNA-PKi) starting 1 h prior to doxorubicin treatment. *MLL*bcr-specific band intensities (EGFP/MLLbcr reporter primers) were normalized to control PCR intensities (mean values for GAPDH, RARα) and expressed relative to doxorubicin-only treatment (without peptide), set to 100%. For primer design see Figure 5a. Data are presented as mean +SEM based on 5 independent experiments. Statistical significance was determined using the two-tailed Wilcoxon matched-pairs signed-rank test and values of p<0.1 are indicated. Panels on the right illustrate reliability of PCR quantification by showing results from control amplifications engaging 50% and 100% template DNA from ctrl samples (H_2O) in 6 independent experiments. PCR products were separated on UltraPure™ Agarose-1000 (Invitrogen/Thermo Fisher Scientific). Stippled line indicates cropping. Uncropped images in Source Data (uncropped images). (**b**) *MLL*bcr rearrangements in presence of DNA-PKi. After nucleofection, K562 reporter cells were cultured for 24 h, mock-treated or treated for 1 h with DNA-PKi, then co-treated for 4 h with DNA-PKi and doxorubicin (2 µM), released in drug-free medium for 72 h, recombination frequencies determined flow cytometrically using a CytoFLEX flow cytometer and relative recombination (rec.) frequencies were determined by normalization to controls (ctrl). Below viabilities according to SSC/FSC-gating are shown. Data are presented as mean +SEM. Statistically significant differences were calculated by two-tailed Mann-Whitney-U test and values of p<0.1 are indicated. Left panels: Recombination measurements after nucleofection with and without 100 µg Ku3 (n=9 samples from 3 independent experiments). Middle panels: Recombination measurements after I-SceI expression with and without DNA-PKi treatment (n=6 samples from 2 independent experiments). Right panels: DNA-PKi effect. Western blot analysis of DNA-PKcs (phospho S2056), DNA-PKcs and Vinculin 4 h after DMSO/etoposide treatment with DNA-PKi treatment starting 1 h earlier. Representative Western blot is shown on top, quantification of DNA-PKcs (phospho S2056) versus DNA-PKcs band intensities below (means of 2 independent experiments, SD). Uncropped Western blot in Source Data (uncropped images). (**c**) NHEJ measurements with Ku3. K562 cells were nucleofected with and without Ku3 (100 µg) as well as NHEJ reporter (10 µg) and I-SceI expression plasmid (10 µg) followed by cultivation for 48 h. Frequencies of EGFP-positive living cells in the population were determined using a CytoFLEX flow cytometer, normalized to the mean of ctrl values (average: 3.4×10^{-2}). SSC/FSC-gating was performed during each recombination experiment as in Supplementary Figure 2c and results depicted below. Data (n=9 samples from 3 independent experiments) are presented as mean +SEM. No statistically significant differences were found by two-tailed Mann-Whitney-U test. Source data are provided as a Source Data file.

Supplementary Figure 6.
Molecular dynamics simulations of Ku3 with the EndoG dimer: Conformational sampling of the interaction of Ku3 with the EndoG dimer.

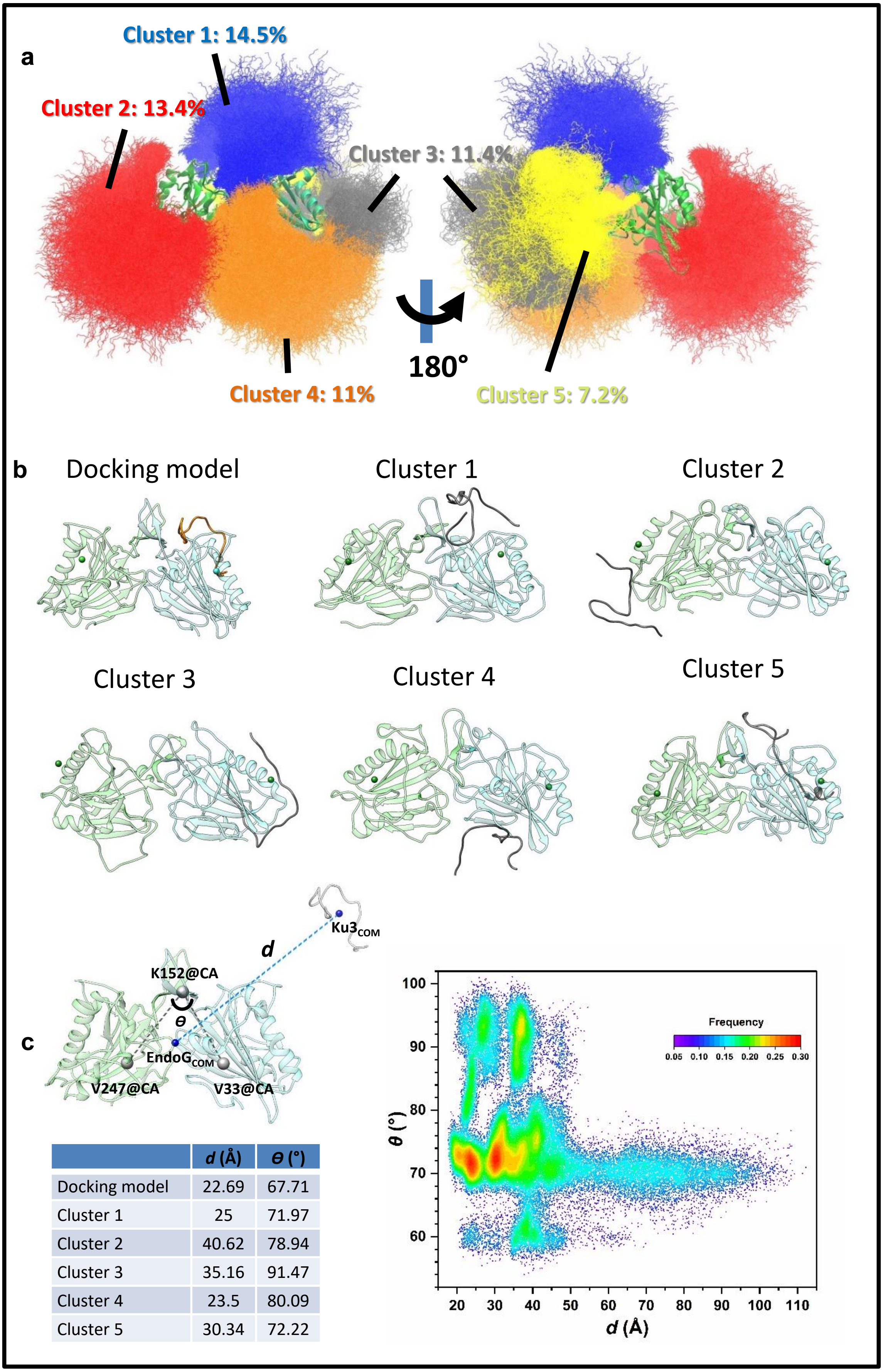

	d (Å)	*θ* (°)
Docking model	22.69	67.71
Cluster 1	25	71.97
Cluster 2	40.62	78.94
Cluster 3	35.16	91.47
Cluster 4	23.5	80.09
Cluster 5	30.34	72.22

continued Supplementary Figure 6.

Molecular dynamics simulations of Ku3 with the EndoG dimer: Conformational sampling of the interaction of Ku3 with the EndoG dimer.

Supplementary Figure 6. Molecular dynamics simulations of Ku3 with the EndoG dimer: Conformational sampling of the interaction of Ku3 with the EndoG dimer. (**a**) Population of each cluster of structures. The percentage is calculated against the total number of frames used for the analysis (total simulation time 6.3 μs). (**b**) Structures of the docking model and representative structures of each cluster. The Ku3 peptide is shown in dark gray, Mg^{2+} is shown as a green sphere. (**c**) Analysis of EndoG/Ku3 COMs distances and EndoG inter-chain fluctuations. Top left: Starting configuration of the EndoG and Ku3 system for the MD simulations. The dashed blue line indicates the distance between the COMs (d) of EndoG and Ku3 (blue spheres). Gray spheres indicate the atoms used for the calculation of the inter-chain angle (θ). Bottom left: d and θ values for the docking model and the representative structures of each cluster. Right: Scatter plot of d vs. θ. The color scale shows the frequency of d/θ pairs.

Supplementary Figure 7. Peptides' motif identification important for the binding to EndoG.

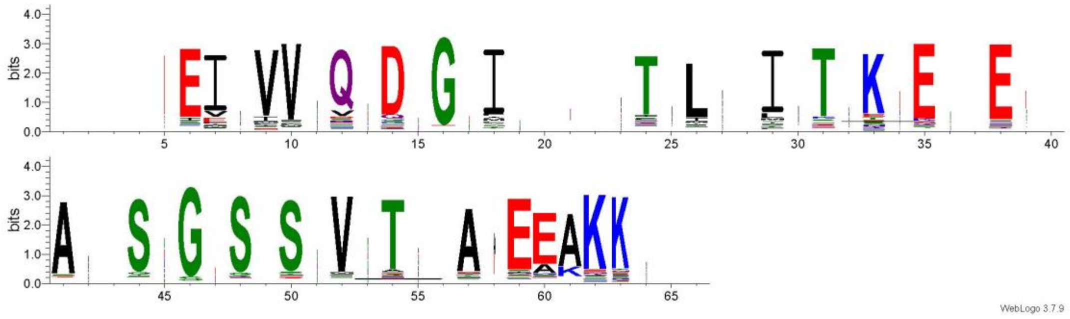

Supplementary Figure 7. Weblogo of the multiple sequence alignment (MSA) of 18960 peptides with an interaction score with EndoG equal or larger than Ku3. The letters represent the conservation of the residues at the given position. Empty positions represent a gap in the MSA. Small letters represent a non-conserved positions in the MSA.

Supplementary Figure 8.
Electrostatic interactions of $Ku3_{E689,K702,D681}$ with EndoG

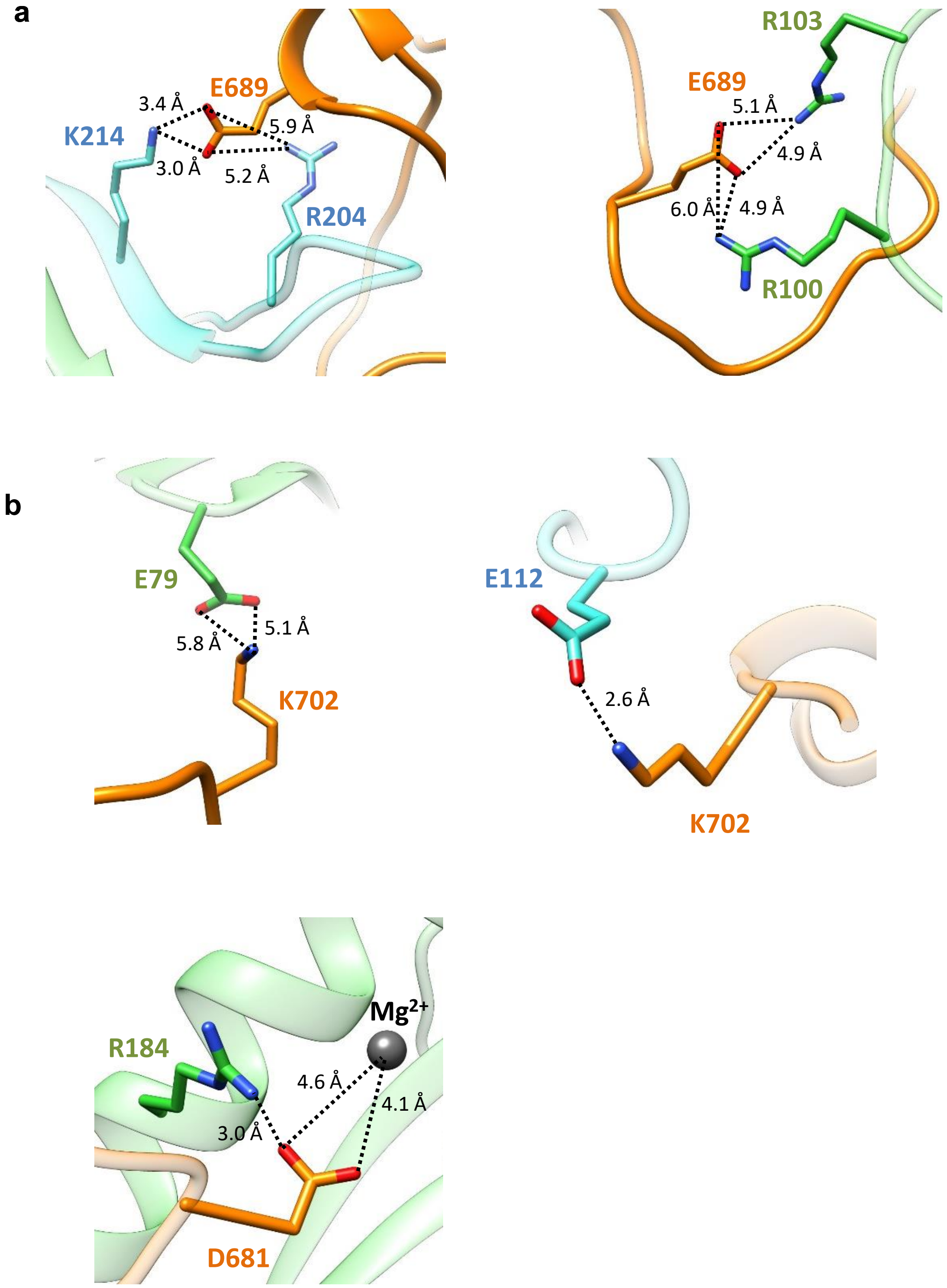

Supplementary Figure 8. Electrostatic interactions of $Ku3_{E689,K702,D681}$ (orange) with EndoG (cyan or light green). Residues in Ku3 are numbered according to the Ku80 sequence. (**a**) Interactions of $Ku3_{E689}$ with $EndoG_{R204,K214}$. (**b**) Interactions of $Ku3_{K702}$ with $EndoG_{E79,E112}$. (**c**) Interactions of $Ku3_{E681}$ with $EndoG_{R184}$ and Mg^{2+} (gray sphere).

Supplementary Figure 9.

Ku3 interferes with the formation of complexes between Ku80 and EndoG during growth.

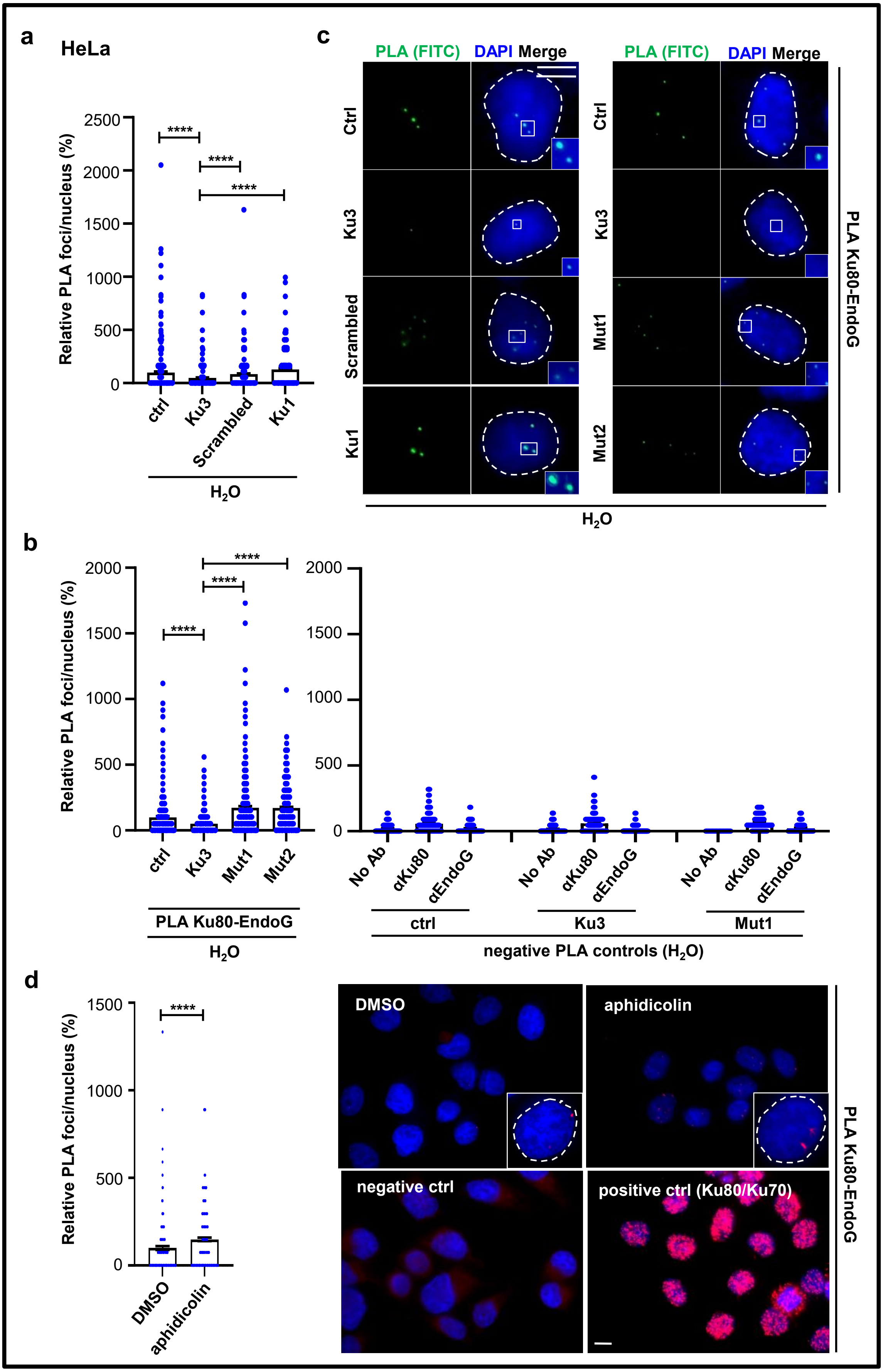

Continued Suppementary Figure 9.
Ku3 interferes with the formation of complexes between Ku80 and EndoG during growth.

Supplementary Figure 9. Ku3 interferes with the formation of complexes between Ku80 and EndoG during growth.
Proximity ligation assay (PLA) was performed on fixed HeLa cells using antibodies against Ku80 and EndoG to detect Ku80-EndoG complex formation. PLA foci numbers were normalized to the means of mock-treated and/or -transfected cells and set to 100% per experiment (average: 1 focus/nucleus). Statistical significance was determined using the Kruskal-Wallis H-test followed by the two-tailed Mann-Whitney U test (****$p<0.0001$). (**a**) Sequence-specific effect of Ku3 on PLA of Ku80-EndoG complexes after mock-treatment. HeLa cells were nucleofected with Ku3, scrambled Ku3, or Ku1 peptide, cultured for 24 h, and mock-treated with H_2O (4 h). Data are presented as mean +SEM from n=604 nuclei for ctrl, n=623 nuclei for Ku3, n=558 nuclei for Scrambled and n=358 nuclei for Ku1 from 4 independent experiments. (**b**) Mutated Ku3 versions do not affect Ku80-EndoG interactions after mock-treatment. HeLa cells were nucleofected with Ku3 or two mutated versions of Ku3 (Mut1 and Mut2), cultured for 24 h and mock-treated with H_2O (4 h). Data are presented as mean +SEM from n=505 nuclei for ctrl, n=545 nuclei for Ku3, n=466 nuclei for Mut1 and n=381 nuclei for Mut2 from 3 independent experiments. PLA control data were performed either without antibodies (no Ab), with anti-Ku80 (αKu80) antibody only or with anti-EndoG (αEndoG) antibody only for cells nucleofected without peptide, with Ku3 or with Mut1 and foci numbers normalized to the average value of PLA Ku80-EndoG ctrl data (100%). Data are presented as mean +SEM (n=103 nuclei for ctrl, no Ab, n=92 nuclei for ctrl, αKu80, n=99 nuclei for ctrl, αEndoG from 4 independent experiments; n=134 nuclei for Ku3, no Ab, n=84 nuclei for Ku3, αKu80, n=133 nuclei for Ku3, αEndoG from 4 independent experiments; n=21 nuclei for Mut1, no Ab, n=53 nuclei for Mut1, αKu80, n=57 nuclei for Mut1, αEndoG from 2 independent experiments). (**c**) PLA image gallery for (a)-(b). Margins of DAPI-stained nuclei are indicated by white stippled lines, and the scale bar represents 10 µm. Insets display highlighted regions at 2-fold magnification. (**d**) Replication stress promotes Ku80-EndoG complex formation. PLA Ku80-EndoG analysis was performed in cells after mock- or aphidicolin-treatment. Left panel: Data are presented as mean +SEM (n=322 nuclei for DMSO, n=344 nuclei for aphidicolin from 2 independent experiments). Right panel: PLA overview images for left panel. Margins of DAPI-stained nuclei are indicated by white stippled lines and the scale bar represents 10 µm. Insets display individual cells with foci at 2.5-fold magnification. Source data are provided as a Source Data file.

Supplementary Figure 10.
EndoG-HT expression in HeLa cells.

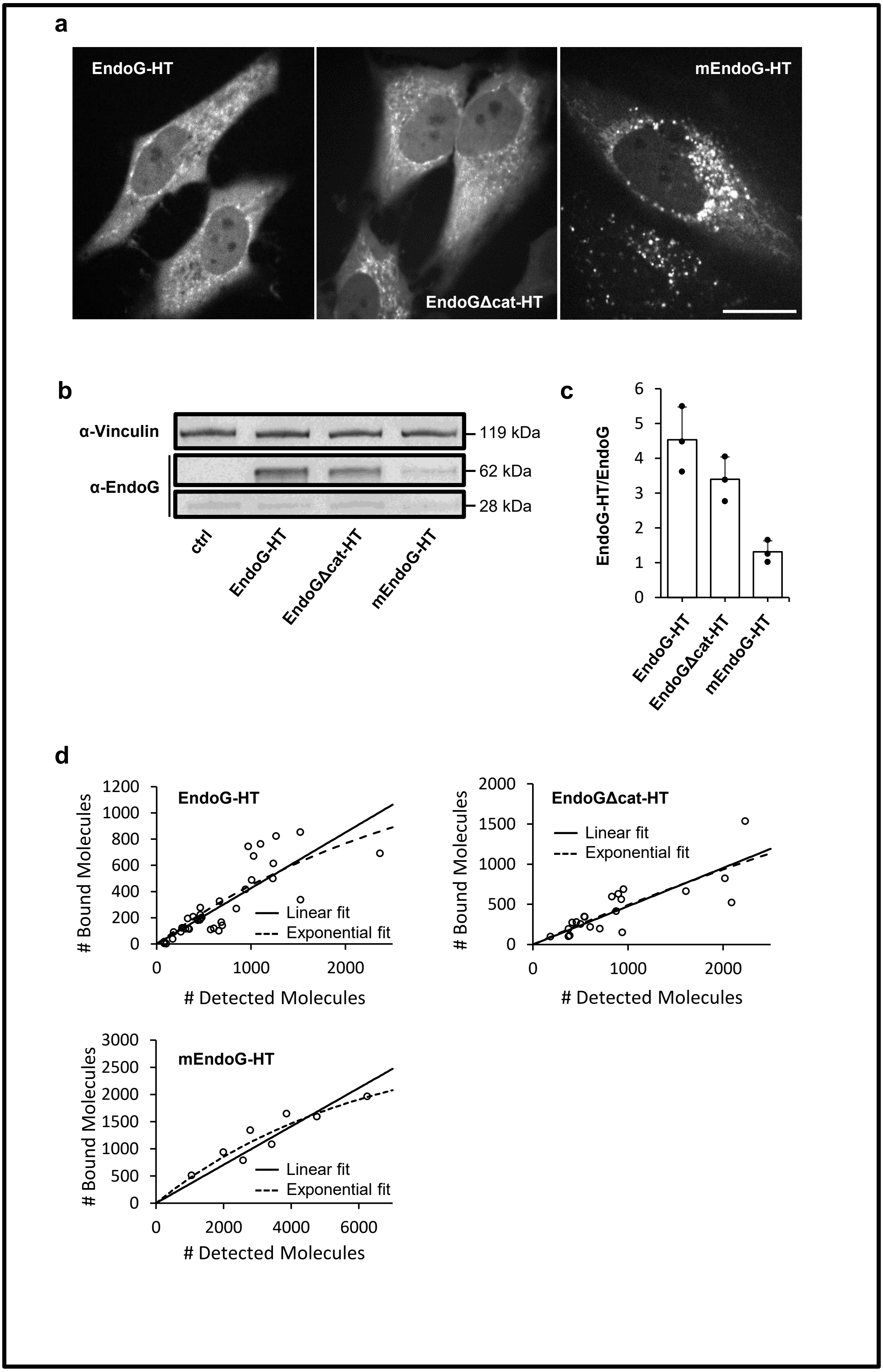

continued Supplementary Figure 10.
EndoG-HT expression in HeLa cells.

Supplementary Figure 10. EndoG-HT expression in HeLa cells. (**a**) HeLa cells stably expressing EndoG-HT variants. EndoG-HT and mEndoG-HT were labeled with HTL-SiR, EndoGΔcat-HT was labeled with HTL-TMR. Representative field of views of >20 cells. Scale bar: 20 µm. (**b**) Western blots of lysates from the cell lines expressing EndoG-HT used for single-molecule tracking, compared with untransduced HeLa (ctrl). Blots were labeled with antibodies against Vinculin and EndoG. Uncropped images in Source Data (uncropped images). (**c**) Mean ratios ± standard deviations of EndoG-HT and endogenous EndoG expression levels (n=3 technical replicates). EndoG-HT and EndoG levels were measured at 62 kDa and 28 kDa, respectively (see panel b). (**d**) Analysis of single-molecule tracking data to assess potential binding saturation due to overexpression shown in (c). The number of bound molecules per movie tracked in at least two consecutive frames of 50 ms frame cycle time was plotted against the total number of detected molecules for EndoG–HT and the two mutants EndoGΔcat–HT and mEndoG–HT, respectively, each labeled with SiR–HTL. A near-linear relation without evidence of saturation (horizontal asymptote) was observed. Source data are provided as a Source Data file. Measurement statistics are given in Supplementary Table 3.

Supplementary Figure 11.
Jump distance distributions of EndoG with three-exponential Brownian diffusion fit.

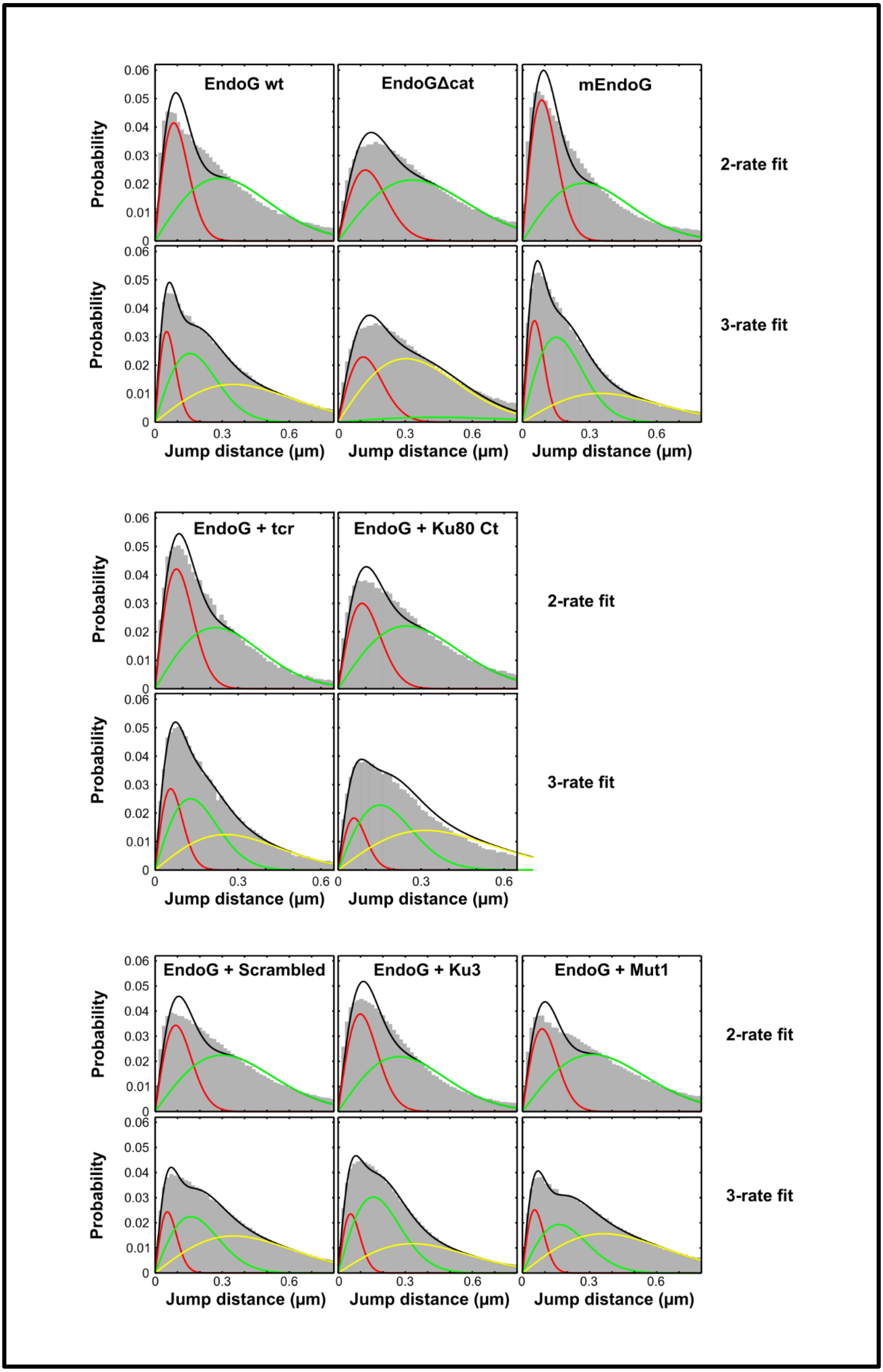

continued Supplementary Figure 11.
Jump distance distributions of EndoG with three-exponential Brownian diffusion fit.

Supplementary Figure 11. Jump distance distributions of EndoG with three-exponential Brownian diffusion fit. Jump distance distributions of EndoG variants and EndoG wt in the presence of the transfection control (tcr), Ku80 Ct, and the Ku3-derived peptides. 2-rate fit and 3-rate fit in comparison for each condition. Red line: diffusion rate 1, green line: diffusion rate 2, yellow line: diffusion rate 3, black line: fit. Source data are provided as a Source Data file. Measurement statistics are given in Supplementary Table 3.

Supplementary Figure 12.
Cumulative jump distance, diffusion coefficients and bound fractions of EndoG and variants.

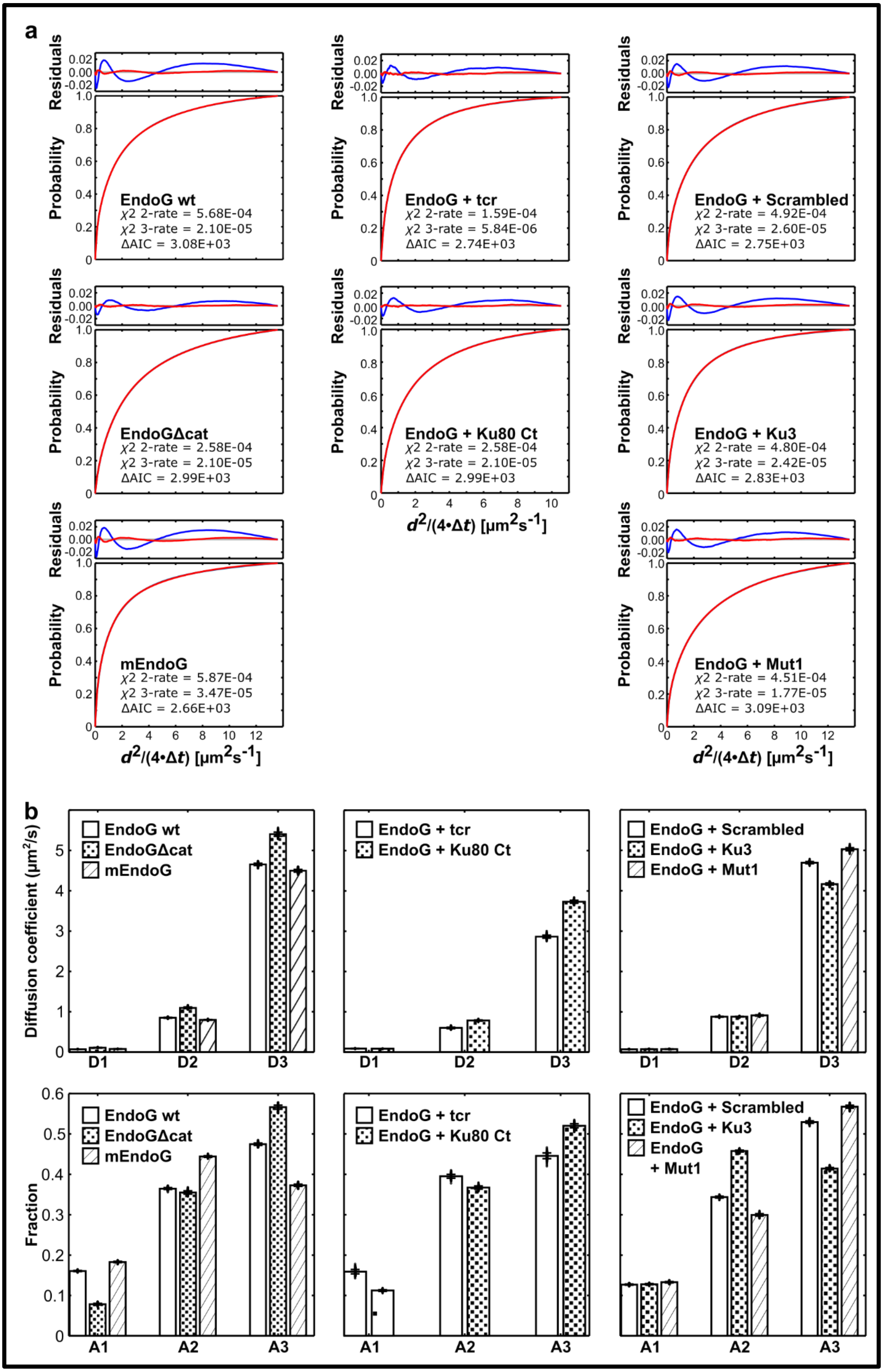

continued Supplementary Figure 12.

Cumulative jump distance, diffusion coefficients and bound fractions of EndoG and variants.

Supplementary Figure 12. Cumulative jump distance, diffusion coefficients and bound fractions of EndoG and variants. (**a**) Bottom graph: Cumulative jump distance distributions with three-component fit (red line) of EndoG variants and EndoG wt in the presence of a transfection control plasmid (tcr), Ku80-Ct, and Ku3-derived peptides. Top graph: Comparison of two-rate fit (blue) and three-rate fit (red) by their deviation from the measurement data (grey). Reduced $\chi 2$ and Akaike information criterion (AIC) support the three-rate model. (**b**) Diffusion coefficients and corresponding fractions extracted from the 3-rate fit in a). The slowest diffusion rate D1 with fraction A1 was used as “bound fraction” in our calculations. Shown values are the mean and SD derived from 500 repetitions of the fit with 80% of the data. Source data are provided as a Source Data file. Measurement statistics are given in Supplementary Table 3.

Supplementary Figure 13.
Residence time measurements.

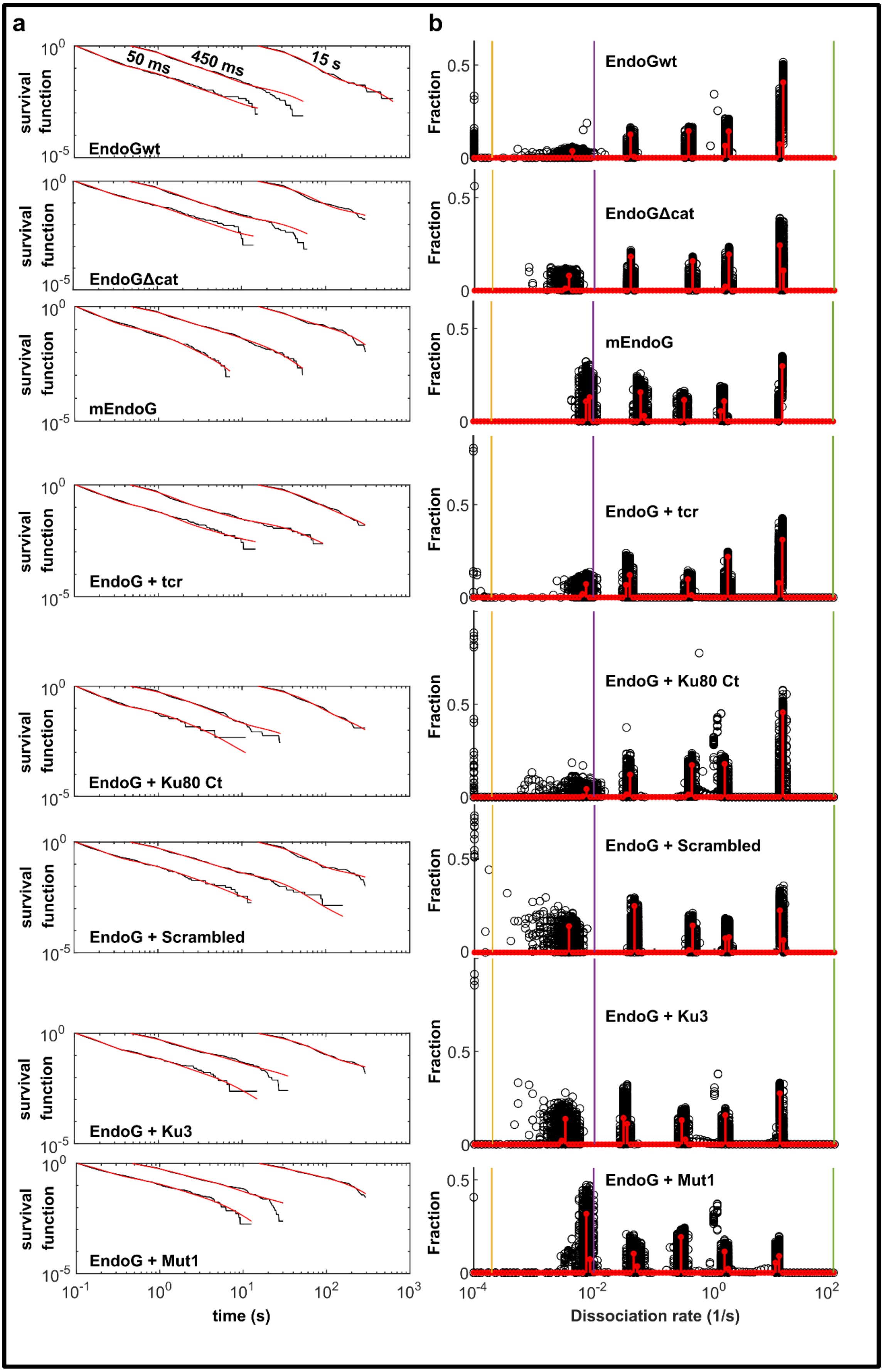

continued Supplementary Figure 13.
Residence time measurements.

Supplementary Figure 13. Residence time measurements. (**a**) Survival time distributions of EndoG variants and EndoG wt in the presence of a transfection control plasmid (tcr), Ku80 Ct and Ku3-derived peptides at time-lapse conditions shown in the top graph. The black line shows raw data, the red line indicates the fit from the inverse Laplace Transformation. (**b**) State spectra of dissociation rates of EndoG variants and EndoG wt in the presence of tcr, Ku80 Ct and Ku3-derived peptides, obtained with GRID using all data (red). As an error estimation, GRID was run 500 times with each run using 80 % of the data (black circles). We included dissociation rates between 0.0002 s^{-1} (yellow line) and 99 s^{-1} (green line) into our calculations of the residence times and corresponding fractions. Dissociation rates below 0.01 s^{-1} (purple line) were defined as long binding, dissociation rates above as short binding. Source data are provided as a Source Data file. Measurement statistics are given in Supplementary Table 3.

Supplementary Tables

Supplementary Table 1. Cloning primers

Primer	Sequence
Primer 1vw	GCGTCTAGAATGGCAGAAATCGGTACTGG
Primer 1 rv	TGGCGCGCCTCAGCCGGAAATCTCGAGCGTCG
Primer 2fw	AATATTGAATTCATGCGGGCGCT
Primer 2rv	ATTAATCTAGAAGCTGCACCGGA
Primer 3fw	AATTCTCGAGATGCCTACAGCTAAAAAATTAAAGGTGAATCCTGCTGAAAAC
Primer 3vr	ATAGGATCCTTAGTCTTTGGGGGCCAG

Supplementary Table 2. EndoG dissociation rates

Construct	Slow dissociation rate (1/s)	Fast dissociation rate (1/s)	Slow fraction (%)	Fast fraction (%)
EndoG wt	0.0046 +/- 0.0014	7.23 +/- 0.48	3.9 +/- 2.1	93.8 +/- 4.2
EndoGΔcat	0.0037 +/- 0.0008	5.48 +/- 0.28	9.4 +/- 1.5	90.5 +/- 2.5
mEndoG	0.0075 +/- 0.0016	5.84 +/- 0.31	21.0 +/- 7.5	76.4 +/- 4.1
EndoG + tcr	0.0068 +/- 0.0017	6.28 +/- 0.5	8.7 +/- 2.7	90.2 +/- 5.9
EndoG + Ku80 Ct	0.0061 +/- 0.0021	6.39 +/- 1.8	5.9 +/- 5.7	92.2 +/- 11.0
EndoG + Scrambled	0.0036 +/- 0.0012	4.75 +/- 0.37	15.0 +/- 4.2	83.5 +/- 8.0
EndoG + Ku3	0.0034 +/- 0.0010	4.38 +/- 0.53	16.4 +/- 3.8	83.1 +/- 6.6
EndoG + Mut1	0.0073 +/- 0.0015	3.1 +/- 0.51	36.9 +/- 8.3	61.3 +/- 5.1

Table legend

Dissociation rates are given as the average rate below (slow dissociation rate) and above (fast dissociation rate) 0.01 s^{-1}, extracted with their corresponding fractions from the dissociation rate spectra in Supplementary Figure 13b.

Supplementary Table 3. Single-molecule tracking measurement statistics

Figure	Statistics
Figure 7b	EndoG wt: n=236,156 jump distances from 32 cells and 3 independent experiments, mEndoG: n=256,407 jump distances from 35 cells and 3 independent experiments, EndoGΔcat: n=154,515 jump distances from 26 cells and 2 independent experiments.
Figure 7e,f	EndoG wt: n=3,879 survival times from 94 cells and 18 independent experiments, mEndoG: n=12,360 survival times from 45 cells and 3 independent experiments, EndoGΔcat: n=3,688 survival times from 64 cells and 10 independent experiments.
Figure 7g	Statistics identical to Figure 7e + Supplementary Figures 13
Figure 7h	Statistics identical to Figure 7b,e + Supplementary Figures 12,13
Supplementary Figure 10d	EndoG-HT: n=36 cells from 17 independent experiments; EndoGΔ-HT: n=21 cells form 8 independent experiments; mEndoG-HT: n=8 cells from 1 experiment;
Supplementary Figure 11	EndoG wt: n=236,156 jump distances from 32 cells and 3 independent experiments, EndoGΔcat: n=154,515 jump distances from 26 cells and 2 independent experiments, mEndoG: n=256,407 jump distances from 35 cells and 3 independent experiments, EndoG + tcr: n=55,329 jump distances from 19 cells and 2 independent experiments, EndoG + Ku80 Ct: n=150,575 jump distances from 26 cells and 3 independent experiments, EndoG + Scramble: n=270,510 jump distances from 40 cells and 3 independent experiments, EndoG + Ku3: n=351,048 jump distances from 34 cells and 3 independent experiments, EndoG + Mut1: n=164,796 jump distances from 36 cells and 3 independent experiments.
Supplementary Figure 12	EndoG wt: n=236,156 jump distances from 32 cells and 3 independent experiments, EndoGΔcat: n=154,515 jump distances from 26 cells and 2 independent experiments, mEndoG: n=256,407 jump distances from 35 cells and 3 independent experiments, EndoG + tcr: n=55,329 jump distances from 19 cells and 2 independent experiments, EndoG + Ku80 Ct: n=150,575 jump distances from 26 cells and 3 independent experiments, EndoG + Scramble: n=270,510 jump distances from 40 cells and 3 independent experiments, EndoG + Ku3: n=351,048 jump distances from 34 cells and 3 independent experiments, EndoG + Mut1: n=164,796 jump distances from 36 cells and 3 independent experiments.
Supplementary Figure 13	EndoG wt: n=3,879 survival times from 94 cells and 18 independent experiments, mEndoG: n=12,360 survival times from 45 cells and 3 independent experiments, EndoGΔcat: n=3,688 survival times from 64 cells and 10 independent experiments, EndoG + tcr: n=2,579 survival times from 100 cells and 11 independent experiments, EndoG + Ku80 Ct: n=1,014 survival times from 93 cells and 12 independent experiments, EndoG + Scramble: n=1,939 survival times from 60 cells and 3 independent experiments, EndoG + Ku3: n=1,475 survival times from 77 cells and 7 independent experiments, EndoG + Mut1: n=1,492 survival times from 48 cells and 5 independent experiments.

Supplementary Methods

Immunoprecipitation

HeLa cells were treated with or without 10 µm aphidicolin for 4 h. For immunoprecipitation cells were washed in ice-cold PBS, harvested in ice-cold PBS, centrifuged and lysed for 30 min on ice in 20 mM Tris, pH 8.0; 150 mM NaCl; 1 mM EDTA; 0.5% NP40; 10U Benzonase; one tablet cOmplete Mini EDTA Protease Inhibitor (Roche, Basel, Switzerland). Lysates were adjusted to equal protein concentrations, and 500 µg total protein was used per IP reaction. Protein extract and Protein G Sepharose (PGS) beads from GE Life Science, Chicago, USA, (10% slurry, washed and resuspended in lysis buffer) were mixed and rotated over night at 4°C to remove components unspecifically binding to PGS. In parallel, antibody-PGS mixtures were rotated at 4°C (1-2 µg polyclonal rabbit antibody directed against Ku80 from Santa Cruz Biotechnology, Dallas, Texas, USA, H-300, sc-9034, per 500 µg protein). Protein-extracts were separated from PGS by centrifugation and the precleared protein extract transferred to the antibody-PGS slurry and rotated over night at 4°C. Following 3 washing steps in lysis buffer and centrifugation, pellets were subjected to SDS-polyacrylamide gel electrophoresis and Western blot analysis. Antibodies used for immunoprecipitation: polyclonal rabbit antibody directed against Ku80 (Santa Cruz Biotechnology, Dallas, Texas, USA, H-300, sc-9034) or control rabbit IgG (Santa Cruz, sc-2027). Primary antibodies used for immunoblotting were: rabbit polyclonal anti-Ku80 (Santa Cruz Biotechnology, H-300, sc-9034, 1/500) and mouse anti-EndoG (Santa Cruz, sc-365359, 1/500). Secondary antibodies (1/10000) used for immunoblotting were: Peroxidase-coupled goat anti-rabbit (Rockland, Pennsylvania, USA) and goat anti-mouse Fcγ (Jackson ImmunoResearch, Newmarket, UK), respectively.

Surface plasmon resonance

Recombinant EndoG (Abbexa, Cambridge, UK) was coupled to the surface of a Series S Sensor Chip CM5 (Cytiva, MA, USA) using standard amine coupling chemistry according to the manufacturer's instructions, with 50 µg/µL EndoG in coupling buffer (10 mM Na-acetate, 10 mM NaCl, pH 5.0). The reference flow cell was activated and blocked under identical conditions but without EndoG immobilization to be used for background subtraction.

Binding experiments were conducted on a Biacore S200 (Cytiva), performing single-cycle measurements in triplicate. The analyte, a C-terminal fragment of Ku80 (amino acids 384–732; Biozol, Hamburg, Germany), was diluted in measurement buffer containing 10 mM HEPES (pH 7.5), 100 mM NaCl, 2 mM $MgCl_2$, and 1 mM DTT to the desired concentrations.

Analyte injections were performed at concentrations of 10, 20, 40, 80, 160, and 320 nM at 25°C, with a flow rate of 20 µL/min, an association phase of 120 s, and a dissociation phase of 120 s. Data were collected at 10 Hz and corrected by subtracting the signal from the reference cell. The surface was regenerated between cycles with 1 M NaCl followed by 10 mM glycine (pH 9.5). The equilibrium dissociation constant K_D was determined using the Biacore S200 evaluation software (Version 1.1) by fitting the data to a one-site binding model.

Methods for live-cell single-molecule measurements

Cell culture

HeLa cells (Sigma-Aldrich/Merck, St. Louis, Missouri, USA) and derived stable cell lines were cultivated for single-molecule measurements in Dulbecco's Modified Eagle Medium (DMEM, 11960085, Gibco™, Thermo Fisher Scientific, Waltham, Massachusetts, USA), supplemented with 10% Fetal Bovine Serum (FBS, F7524, Sigma-Aldrich/Merck), 1% Sodium Pyruvate (11360039, Gibco™, Thermo Fisher Scientific), 1% GlutaMAX™ Supplement (35050038, Gibco™, Thermo Fisher Scientific) and 1% MEM solution of non-essential amino acids (11140035, Gibco™, Thermo Fisher Scientific). The cells were grown in 6 cm culture dishes (83.3901, Sarstedt, Nümbrecht, Germany) and passaged twice weekly upon reaching 60 – 80 % confluency. Cells were incubated at 37 °C and 5% CO_2.

Stable cell line generation

Cloning of expression plasmids

<u>pLVTO-EndoG-Halo</u>

The HaloTag® sequence[4] from a DNA template[5] was PCR amplified with the Q5 High-Fidelity DNA Polymerase (New England Biolabs, Frankfurt, Germany), following the manufacturer's standard protocol. The amplification was performed with an annealing temperature of 72 °C and the primers 1fw and 1rv (Supplementary Table 1). All primers used in the cloning process for single-molecule experiments were custom orders from Sigma-Aldrich/Merck. Post-amplification, the PCR product was purified using the Monarch PCR & DNA Cleanup Kit (New England Biolabs) according to their standard protocol and the sample eluted in water. The DNA fragment ends were digested with the restriction enzymes XbaI (New England Biolabs) and AscI (New England Biolabs), and ligated to the pLV-tetO-Oct4 backbone gifted from Konrad Hochedlinger[6](#19766, Addgene, Watertown, Massachusetts, USA), which had been modified to incorporate XbaI and AscI restriction sites. This backbone not only allows lentiviral transduction, but also ensures low expression levels of the protein-of-interest in the target cells through leaky expression by the repressed promotor system. The ligation product served as the plasmid backbone for the subsequent cloning step.

Following PCR amplification of the *ENDOG* wild-type (EndoG wt) cDNA sequence according to the protocol above, but adding GC enhancer and using the primers 2fw and 2rv (Supplementary Table 1), both the purified EndoG wt fragment and plasmid backbone were digested with the restriction enzymes EcoRI (New England Biolabs) and XbaI and ligated, thereby fusing the HaloTag® to the C-terminus of EndoG wt (EndoG-HT).

<u>pLVTO-EndoGΔcat-Halo</u>

The cDNA sequence of *ENDOG* mutant EndoGΔcat carrying the point mutations R133A (CGC → GCC), H141A (CAC →GCC), H148A (CAC → GCC), R184A (CGC → GCC), and R188A (CGC →GCC) was purchased from Invitrogen GeneArt (Thermo Fisher Scientific). The plasmid was amplified, digested and ligated as described above for EndoG wt.

<u>pLVTO-mEndoG-Halo</u>

The cDNA sequence of the monomeric *ENDOG* mutant (mEndoG) with the point mutation P199E (CCA → GAG) was generated at GeneArt (Thermo Fisher Scientific). Following amplification, the fragment was cloned into the backbone as described for EndoG wt.

pEGFP-NLS-Ku80Ct

XRCC5 cDNA encoding the Ku80 Ct (amino acids 592-709) was amplified via PCR from our pEGFP-C1-FLAG-KU80 plasmid gifted from Steve Jackson [7](#46958, Addgene) using the Q5 High-Fidelity DNA Polymerase (New England Biolabs) following the manufacturer's standard protocol and primers 3fw and 3rv (Supplementary Table 1) with a hybridization temperature of 70 °C. An over-hang of primer 3fw incorporated the Ku80 NLS motif "PTAKKLK"[8].

The NLS-Ku80 Ct fragment was inserted into the plasmid pEGFP-C1-FLAG through enzymatic digestion of both the insert and backbone with XhoI and BamHI, followed by ligation. This process removed the C1-FLAG sequence, resulting in the final construct, pEGFP-NLS-Ku80Ct.

All plasmid sequences of individual clones were verified by Sanger sequencing (Mycrosynth AG, Balgach, Switzerland).

Cell transduction

Handling of lentivirus and cells during the transduction process and subsequent cell handling were according to current European and German regulations.

LentiX-293T (Clontech Laboratories, Palo Alto, California, USA) cells were cultured until they reached 80% confluency, then transfected with the virus packaging plasmids psPAX2 (7.5 µg, #12260, Addgene, Watertown, Massachusetts, USA) and pMD2.G (2.5 µg, #12259, Addgene), alongside with 10 µg of the expression plasmid of interest, using the JetPrime kit (Polyplus, Sartorius, Göttingen, Germany). Following transfection, the cells were incubated at 37 °C, 5% CO_2 for 2 days.

Subsequently, we harvested the virus-containing supernatant and filtered it through a 0.45 µm Whatman™ membrane filter (Cytiva, Marlborough, Massachusetts, USA). We added 1 ml of filtered solution to HeLa cells, which had been cultured to 60 % confluency in a 6-well dish, incubated the cells at 37 °C and 5% CO_2. Subsequently, we washed the cells with 3 ml PBS and treated them with 500 µl of 0.05% Trypsin-EDTA (25200056, Gibco™, Thermo Fisher Scientific) for 4 min to facilitate detachment. The reaction was terminated with 3.5 ml DMEM, and 3.5 ml cell suspension were transferred into a 10 cm culture dish, prefilled with 6.5 ml DMEM. To confirm transduction, we transferred 0.5 ml of the cell suspension into a microscopy dish (81158, ibidi®, Gräfelfing, Germany), containing 3.5 ml DMEM.

After one day of incubation, we labeled the cells in the microscopy dish with 1 ml of 5 µM HaloTag® TMR ligand (HTL-TMR, Promega, Madison, Wisconsin, USA) for 30 min. After washing with PBS, we replaced the medium with 2 ml of OptiMEM (Gibco™, Thermo Fisher Scientific), and assessed protein expression using a confocal spinning disc microscope (Axio Observer D1, Carl Zeiss Microscopy, Jena, Germany, with a CSU10 Confocal Scanner Unit, Yokogawa Electric, Tokyo, Japan).

To ensure expression of mEndoG in all cells, we labeled the HeLa mEndoG-HT cell line with 1.25 µM HTL-TMR ligand for 15 min, and isolated cells that exhibited TMR signal using fluorescence-activated cell sorting at the *Core Facility Cytometry*, Ulm University.

Western blot

When testing for overexpression in single-molecule tracking cell lines, cell lysates were created from scraped HeLa cells stably expressing EndoG-HT, EndoGΔcat-HT, or mEndoG-HT. The cells were twice centrifuged (3 min, 456 x g, 4 °C) and resuspended in ice-cold RIPA buffer (Sigma-Aldrich/Merck) with the addition of a protease and phosphatase inhibitor. Subsequently, the cell suspensions were incubated on ice for 20 min during which they were vortexed every 5 min. Afterwards, the lysed cells were centrifuged (20 min, 17949 x g, 4°C) and the supernatant containing the cellular proteins was collected. Protein concentrations were determined with the BCA Protein Assay Kit (Thermo Fisher Scientific). Protein samples were subjected to denaturing SDS-polyacrylamide gel electrophoresis using self-poured 12% poly-acrylamide gels and PageRuler Prestained (Thermo Fisher Scientific) followed by Western transfer. The membrane was labelled with the primary antibody over night at 4 °C and with the goat anti-mouse secondary antibody (Invitrogen/Thermo Fisher Scientific, Waltham, Massachusetts, USA) for 60 min at room temperature. For primary antibodies, we used anti-EndoG (Mouse monoclonal, sc-365359, Santa Cruz) and anti-Vinculin (mouse monoclonal, V9131, Sigma-Aldrich). Protein bands were visualized with BCIP/NBT-Blue Liquid Substrate System (sc-291929, Santa Cruz) and imaged with a ChemiDoc MP Imaging System (Bio-Rad Laboratories).

Transient transfection

For single-molecule measurements investigating the impact of Ku80-Ct on EndoG wt binding, we transiently transfected the HeLa EndoG-HT cell line with either the pEGFP-NLS-Ku80Ct plasmid or the control plasmid pmaxGFP (Lonza). We seeded cells into the microscopy dish two days prior to the measurements and allowed them to reach 80% confluency.

Twenty-five hours before the measurements, we mixed 200 µL jetPRIME buffer (Polyplus, Sartorius, Göttingen, Germany) with 2 µg of plasmid, vortexed for 10 s, and added 4 µl jetPRIME reagent (Polyplus, Sartorius). After a 1 s vortex pulse and a brief centrifugation, we incubated the mixture at room temperature for 10 min, added the mix dropwise onto the cells and incubated them for 5 h at 37 °C and 5% CO_2. After incubation, we washed the cells with 1 ml PBS and added 2 ml of fresh medium.

Nucleofection

To assess the impact of the peptides Ku3 and Mut1 on EndoG wt binding, we engaged N-terminally TAMRA labeled peptides from Pepscan/Biosynth (Staad, Switzerland), dissolved the lyophilized peptides in distilled water and stored them at -20 °C.

Cells were cultivated on a 6 cm tissue culture dish until reaching 60-80% confluency on the day of measurements, followed by a wash in 2 ml PBS and trypsinization. One million cells were centrifuged at 168 – 233 x g for 3 – 6 min and resuspended in 82 µl nucleofection buffer mixed

with 18 µl of Supplement 1 from the Lonza Nucleofection Kit R (Lonza, Basel, Switzerland). We added the appropriate volume of peptide solution to achieve a peptide concentration of 52 µM. We transferred the mixture to a nucleofection cuvette and ran the I-013 High Expression Program of the Amaxa® Nucleofector® II (Lonza).

Following nucleofection, we added 500 µl of DMEM to the cells and transferred the mix to a microscopy dish, prefilled with 1.5 ml of DMEM. After 2 h of incubation at 37 °C with 5% CO_2, we controlled for successful nucleofection and nuclear localization of TAMRA-labeled peptides. We imaged them on a confocal spinning disc microscope at an excitation wavelength of 532 nm, and compared them to cells nucleofected with an equal volume of water. We applied the same protocol two hours before labeling cells for our single-molecule measurements.

Single-molecule measurement

Cell preparation

When no additional preparations, such as transient transfection, were required, cells were seeded into microscopy dishes one to two days prior to measurements. For diffusion measurements of the HeLa EndoG-HT cell line transfected with either pEGFP-NLS-Ku80Ct or pmaxGFP, 35 mm glass bottom dishes from ibidi® (Gräfelfing, Germany) were used. All other single-molecule measurements were conducted on DeltaT™ dishes (Bioptechs, Butler Pennsylvania, USA).

For time-lapse measurements, we prepared 30 pM of SiR[9] HaloTag ligand (HTL, provided by Kay Johnsson, MPI Heidelberg) in 1 ml of DMEM and incubated the cells with the dye for 15 min. For diffusion measurements, we labeled the cells with 30 nM PA-JF646 HTL (HHMI Janelia Research Campus, Ashburn, Virginia, USA) for 30 min. After labeling, we washed the cells with 1 ml PBS and incubated them in 2 ml of fresh DMEM for at least 30 min prior to measurements. Before imaging, we washed the cells three times with 1 ml PBS and added 2 ml OptiMEM (Gibco™, Thermo Fisher Scientific).

Microscope

We conducted single-molecule measurements using a single-camera custom-built fluorescence microscope[10] in combination with a highly inclined and laminated optical laser sheet (HILO)[11] to enhance signal-to-noise conditions (“Setup 1”). The microscope was equipped with a 405 nm laser (Laser MLD, 299 mW, Solna, Sweden), a 488 nm laser (IBEAM-SMART-488-S-HP, Toptica Photonics, Gräfelfing, Germany), and a 638 nm laser (IBEAM-SMART-640S, 150 mW, Toptica, Photonics).

Diffusion measurements of the HeLa EndoG-HT cell line transiently transfected with pEGFP-NLS-Ku80Ct or pmaxGFP were performed on a custom-built inverted microscope equipped with a beam splitter (F48-644, AHF analysentechnik, Tübingen, Germany), which divides the emission beam path at 643 nm, thereby separating the single-molecule signal from the GFP signal (“Setup 2”). The emission light passing the beam splitter was filtered using a combination of an emission filter and a notch filter (F67-532 and F40-074, AHF analysentechnik). Emission light above 634 nm was detected by a Prime BSI sCMOS camera (Teledyne Scientific Imaging GmbH), while light below 643 nm was captured by a Kinetix 22 sCMOS camera (Teledyne Technologies, Thousand

Oaks, California, USA). The effective pixel size after 2x2 binning was 130 nm for both cameras. The microscope included a 405 nm laser (Cobolt 06-MLD 405 nm, Hübner Photonics, Kassel, Germany) along with an absorptive ND filter (NE10A, Thorlabs, Bergkirchen, Germany), a 488 nm laser (Cobolt 06-MLD 488 nm, Hübner Photonics, Kassel, Germany), and a 638 nm laser (Omicron Luxx Laser, Omicron-Laserage Laserprodukte, Rodgau, Germany). The laser profile was modified from a Gaussian shape to a flat top using a diffractive beam shaper (piShaper 6_6_VIS, AdlOptica, Berlin, Germany)

Both microscopes were equipped with a heating source, heating the cell medium to 37 °C.

Diffusion measurements

To quantify the fractions of bound and free EndoG molecules in the nucleus, we acquired movies with a 10 ms exposure time per frame to capture fast diffusion dynamics.

On Setup 1, the protein-coupled PA-JF646 dye was activated using a 1 ms pulse of the 405 nm laser while simultaneously excited with a 10 ms pulse of the 638 nm laser. Due to the exposure and read-out time, the effective camera integration time resulted in 11.7 ms per frame.

On Setup 2, we continuously activated the PA-JF646 dye with 405 nm and excited it with 638 nm in 10 ms pulses. Before each measurement, we selected cells that showed a GFP signal upon excitation with 488 nm, to verify successful plasmid transfection. The camera integration time was 10 ms.

We recorded movies with 20,000 frames each.

Time-lapse measurements

To assess EndoG binding events, we recorded continuous movies with a camera integration time of 51.7 ms per frame on Setup 1. The SiR dye was excited with the 638 nm laser. To capture long binding events that might otherwise experience photobleaching, we recorded time-lapse movies with dark intervals of 450 ms and 15 s between frames, respectively.

Analysis

Tracking

To analyze movies, we imported TIFF files into the TrackIt software[12] for single-molecule localization and track generation. We confined the tracking region to the cell nucleus.

For diffusion measurements with a frame time of 10 or 11.7 ms, we employed a tracking radius of 5 pixels (0.83 µm), with the minimum track length set to two frames, and a maximum of one gap frame allowed. For residence time measurements, different time-lapse conditions resulted in varying jump distances between frames due to the underlying motion of chromatin. We therefore adjusted the tracking radius as follows: to 1.2 pixels (0.2 µm) for 51.7 ms continuous measurements, 1.5 pixels (0.25 µm) for 450 ms time-lapse measurements and 3 pixels (0.5 µm)

for 15 s time-lapse measurements. The criteria of a minimum track length of two frames and one gap frame were maintained across all time-lapse conditions.

Diffusion coefficients and bound fraction

We generated cumulative survival time distributions from the tracks of our diffusion measurements. To avoid overrepresentation of immobile molecules, we limited the data to the first five jumps per track. We extracted diffusion rates from the survival time distributions by fitting a Brownian diffusion model[13]. The calculation of chi-squared and the Akaike information criterion (AIC)[14] for a two-state and a three-state diffusion model supported a three-state model for our data, causing us to continue with the latter. We calculated mean and standard deviation from 500 resamplings using random 80% subsets of the full dataset to assess uncertainty. We assumed that the slowest diffusion rate D_1 and its corresponding fraction F_1 represent the overall bound fraction of EndoG.

Binding times and overall long bound fraction

We extracted binding times of EndoG molecules from the survival time histograms of the time-lapse data using the analysis tool GRID[10,15], which performs an inverse Laplace transformation and provides amplitudes for a predefined number of dissociation rates. We selected a total of 100 dissociation rates over the range of 10^{-4} – 100 s^{-1}. Additionally, we conducted 500 resamplings, each consisting of 80% of the dataset, to estimate uncertainty of the dissociations rate spectrum. We removed extreme outliers below 0.0002 s^{-1}. We identified the peak corresponding to dissociation rates below 0.01 s^{-1} as representing long bound EndoG, with the dissociation rate denoted as $k_{off,l,}$ and the long bound fraction as $f_{b,l}$. Peaks with faster dissociation rates were pooled as the short binding fraction $f_{b,s}$. We calculated the long binding time as $t_l = 1/k_{off,l}$. We calculated the overall long bound fraction f_b using the long bound fraction $f_{b,l}$ and the overall fraction F_1 with the equation $f_b = f_{b,l} * F_1$.

Supplementary References

1. Eberle, J. *et al*. A Fibrinogen Alpha Fragment Mitigates Chemotherapy-Induced MLL Rearrangements. *Front. Oncol*. **11**, 689063 (2021).

2. Akyüz, N. *et al*. DNA substrate dependence of p53-mediated regulation of double-strand break repair. *Mol. Cell. Biol*. **22**, 6306-6317 (2002).

3. Bennardo, N., Cheng, A., Huang, N. & Stark, J.M. Alternative-NHEJ is a mechanistically distinct pathway of mammalian chromosome break repair. *PLoS Genet*. **4**, e1000110 (2008).

4. Los, G. V. *et al*. HaloTag: a novel protein labeling technology for cell imaging and protein analysis. *ACS Chem. Biol*. **3**, 373-382 (2008).

5. Agarwal, H., Reisser, M., Wortmann, C. & Gebhardt, J. C. M. Direct Observation of Cell-Cycle-Dependent Interactions between CTCF and Chromatin. *Biophys. J*. **112**, 2051-2055 (2017).

6. Stadtfeld, M., Maherali, N., Breault, D.T. & Hochedlinger, K. Defining molecular cornerstones during fibroblast to iPS cell reprogramming in mouse. *Cell Stem Cell*. **2**, 230-240 (2008).

7. Britton, S., Coates, J. & Jackson, S.P. A new method for high-resolution imaging of Ku foci to decipher mechanisms of DNA double-strand break repair. *J. Cell Biol*. **202**, 579-595 (2013).

8. Koike, M. Dimerization, translocation and localization of Ku70 and Ku80 proteins. *J. Radiat. Res*. **43**, 223-236 (2002).

9. Lukinavičius, G. *et al*. A near-infrared fluorophore for live-cell super-resolution microscopy of cellular proteins. *Nat. Chem*. **5**, 132-139 (2013).

10. Huynh, D. *et al*. Effective in vivo binding energy landscape illustrates kinetic stability of RBPJ-DNA binding. *Nat. Commun*. **16**, 1259 (2025).

11. Tokunaga, M., Imamoto, N. & Sakata-Sogawa, K. Highly inclined thin illumination enables clear single-molecule imaging in cells. *Nat. Methods* **5**, 159-161 (2008).

12. Kuhn, T., Hettich, J., Davtyan, R. & Gebhardt J. C. M. Single molecule tracking and analysis framework including theory-predicted parameter settings. *Sci. Rep*. **11**, 9465 (2021).

13. Kuhn, T. *et al*. Single-molecule tracking of Nodal and Lefty in live zebrafish embryos supports hindered diffusion model. *Nat. Commun*. **13**, 6101 (2022).

14. Coßmann, J. *et al*. Increasingly efficient chromatin binding of cohesin and CTCF supports chromatin architecture formation during zebrafish embryogenesis. *Nat. Commun*. **16**, 1833 (2025).

15. Reisser, M. *et al*. Inferring quantity and qualities of superimposed reaction rates from single molecule survival time distributions. *Sci. Rep*. **10**, 1758 (2020).
