# Supplementary material for: Discovery of an Endonuclease G-inhibitory Ku80-peptide protecting against leukemogenic rearrangements at the MLL breakpoint cluster
Source: Nat Commun. 2026 Apr 17;17:3562. doi: 10.1038/s41467-026-72034-2 (PMC13086865; doi:10.1038/s41467-026-72034-2)
Supplement: Supplementary file 2 — Description of Additional Supplementary Files [file 41467_2026_72034_MOESM2_ESM.pdf]

Description of Additional Supplementary Data Files

File Name: Supplementary Data 1
Description: initial configuration of replica 1

File Name: Supplementary Data 2
Description: final configuration 1 of replica 1

File Name: Supplementary Data 3
Description: final configuration 2 of replica 1

File Name: Supplementary Data 4
Description: final configuration 3 of replica 1

File Name: Supplementary Data 5
Description: initial configuration of replica 2

File Name: Supplementary Data 6
Description: final configuration 1 of replica 2

File Name: Supplementary Data 7
Description: final configuration 2 of replica 2

File Name: Supplementary Data 8
Description: final configuration 3 of replica 2

File Name: Supplementary Data 9
Description: initial configuration of replica 3

File Name: Supplementary Data 10
Description: final configuration 1 of replica 3

File Name: Supplementary Data 11
Description: final configuration 2 of replica 3

File Name: Supplementary Data 12
Description: final configuration 3 of replica 3
