# Supplementary material for: Discovery of an Endonuclease G-inhibitory Ku80-peptide protecting against leukemogenic rearrangements at the MLL breakpoint cluster
Source: Nat Commun. 2026 Apr 17;17:3562. doi: 10.1038/s41467-026-72034-2 (PMC13086865; doi:10.1038/s41467-026-72034-2)
Supplement: Supplementary file 15 — Reporting Summary [file 41467_2026_72034_MOESM15_ESM.pdf]

nature portfolio

NCOMMS-25-22734B,
Corresponding author(s): Lisa Wiesmüller
J. Christof M. Gebhardt
Elsa Sanchez-Garcia

Last updated by author(s): 2026/03/14

Reporting Summary

Nature Portfolio wishes to improve the reproducibility of the work that we publish. This form provides structure for consistency and transparency in reporting. For further information on Nature Portfolio policies, see our Editorial Policies and the Editorial Policy Checklist.

Statistics

For all statistical analyses, confirm that the following items are present in the figure legend, table legend, main text, or Methods section.

n/a	Confirmed	
☐	☒	The exact sample size (*n*) for each experimental group/condition, given as a discrete number and unit of measurement
☐	☒	A statement on whether measurements were taken from distinct samples or whether the same sample was measured repeatedly
☐	☒	The statistical test(s) used AND whether they are one- or two-sided<br>*Only common tests should be described solely by name; describe more complex techniques in the Methods section.*
☒	☐	A description of all covariates tested
☒	☐	A description of any assumptions or corrections, such as tests of normality and adjustment for multiple comparisons
☐	☒	A full description of the statistical parameters including central tendency (e.g. means) or other basic estimates (e.g. regression coefficient) AND variation (e.g. standard deviation) or associated estimates of uncertainty (e.g. confidence intervals)
☐	☒	For null hypothesis testing, the test statistic (e.g. *F*, *t*, *r*) with confidence intervals, effect sizes, degrees of freedom and *P* value noted<br>*Give P values as exact values whenever suitable.*
☒	☐	For Bayesian analysis, information on the choice of priors and Markov chain Monte Carlo settings
☒	☐	For hierarchical and complex designs, identification of the appropriate level for tests and full reporting of outcomes
☒	☐	Estimates of effect sizes (e.g. Cohen's *d*, Pearson's *r*), indicating how they were calculated

Our web collection on statistics for biologists contains articles on many of the points above.

Software and code

Policy information about availability of computer code

Data collection	images BZ-9000 Keyence analyzer and BZ-II Analyser 2.1 software (Keyence, Osaka, Japan); Zeiss fluorescence microscope Axio Observer 3/5/7 KMAT and ZEN Blue software 3.1 (Carl Zeiss AG, Oberkochen, Germany); ChemiDocTM MP Imaging System and Image Lab 5.2.1 to 6.0.1 (Bio-Rad Laboratories, CA, USA); flow cytometry: FACSCalibur™ and BD Cell QuestTM Pro 5.2.1 (BD Biosciences, San Jose, California, USA); CytoFLEX B3-R1-V0 flow cytometer and CytExpert, Version 2.5.0.77 (Beckman Coulter, Brea, California, USA); Molecular modeling: Swiss-Model server (https://swissmodel.expasy.org/); PPI-Detect; CABS-Dock; GROMACS.
Data analysis	images: BZ-II Analyzer 2.1 software (Keyence, Osaka, Japan); ZEN Blue software 3.1 (Carl Zeiss AG, Oberkochen, Germany); Image Lab 5.2.1 to 6.0.1 (Bio-Rad Laboratories); flow cytometry: BD Cell QuestTM Pro 5.2.1 (BD BiosciencesA), CytExpert, Version 2.5.0.77 (Beckman Coulter); statistics: GraphPad Prism Software 8; 9; 10 (GraphPad Software Inc., CA, USA); single-molecule tracking: Matlab R2023a (The MathWorks Inc., MA, Natick); TrackIt (Kuhn et al. Sci Rep 11, 9465 (2021), https://gitlab.com/GebhardtLab/TrackIt, https://doi.org/10.5281/zenodo.7092296);Molecular modeling: VMD; CONAN; GROMACS suite tools; OriginLab 2022b

For manuscripts utilizing custom algorithms or software that are central to the research but not yet described in published literature, software must be made available to editors and reviewers. We strongly encourage code deposition in a community repository (e.g. GitHub). See the Nature Portfolio guidelines for submitting code & software for further information.

Data

Policy information about availability of data

All manuscripts must include a data availability statement. This statement should provide the following information, where applicable:

- Accession codes, unique identifiers, or web links for publicly available datasets
- A description of any restrictions on data availability
- For clinical datasets or third party data, please ensure that the statement adheres to our policy

Data availability statement has been introduced before the list of References and at the beginning of the Methods section, namely: The data of this work can be found in the Source Data files and will be made available upon request without restrictions.

Research involving human participants, their data, or biological material

Policy information about studies with human participants or human data. See also policy information about sex, gender (identity/presentation), and sexual orientation and race, ethnicity and racism.

Reporting on sex and gender	n/a
Reporting on race, ethnicity, or other socially relevant groupings	n/a
Population characteristics	n/a
Recruitment	n/a
Ethics oversight	n/a

Note that full information on the approval of the study protocol must also be provided in the manuscript.

Field-specific reporting

Please select the one below that is the best fit for your research. If you are not sure, read the appropriate sections before making your selection.

☒ Life sciences ☐ Behavioural & social sciences ☐ Ecological, evolutionary & environmental sciences

For a reference copy of the document with all sections, see nature.com/documents/nr-reporting-summary-flat.pdf

Life sciences study design

All studies must disclose on these points even when the disclosure is negative.

Sample size	Experimental sample size estimates were based on our previous related works, Gole et al 2015 Oncogene, Gole et al 2018 Leukemia and Eberle et al Front Oncol, in particular.
Data exclusions	No data were excluded, unless technical problems became apparent (e.g. contamination) or quality controls failed (e.g. saturation). Single-molecule tracking: Movies were excluded from analysis if drift was detected or labeling was too dense for tracking; extreme outliers below 0.0002 s^(-1) were excluded from GRID results;
Replication	Unless otherwise stated, all relevant experiments were performed at least three times. Several scientists obtained consistent experimental results over a period of years.
Randomization	Allocation of samples into experimental groups was random, namely as split samples from one culture of the experimental day.
Blinding	Experiments were not blinded, but several scientists independently obtained consistent results over the years of data accrual.

Reporting for specific materials, systems and methods

We require information from authors about some types of materials, experimental systems and methods used in many studies. Here, indicate whether each material, system or method listed is relevant to your study. If you are not sure if a list item applies to your research, read the appropriate section before selecting a response.

Materials & experimental systems

n/a	Involved in the study	
☐	☒	Antibodies
☐	☒	Eukaryotic cell lines
☒	☐	Palaeontology and archaeology
☒	☐	Animals and other organisms
☒	☐	Clinical data
☒	☐	Dual use research of concern
☒	☐	Plants

Methods

n/a	Involved in the study	
☒	☐	ChIP-seq
☐	☒	Flow cytometry
☒	☐	MRI-based neuroimaging

Antibodies

Antibodies used

anti-Ku80 (Rabbit polyclonal, H-300, sc-9034, Santa Cruz, Western blot (WB): 1/500), anti-Ku80 (Rabbit monoclonal, S.669.4, Invitrogen/Thermo Fisher Scientific, immunofluorescence microscopy (IF): 1/400, PLA: 1/300), anti-Flag M2 (mouse monoclonal, F1804, Sigma-Aldrich/Merck, WB: 1/1000), anti-Flag M2-Peroxidase (HRP, mouse monoclonal, A8592, Sigma-Aldrich/Merck, WB: 1/1000), anti-γH2AX Ser139 (Mouse, monoclonal, Clone JBW 301, 05-636, Merck Millipore, WB and IF: 1/1000), anti-EndoG (mouse monoclonal, B-2, sc-365359, Santa Cruz, WB: 1/500, IF: 1/1000, PLA: 1/300), anti-DNA-PKcs phospho S2056 (Rabbit, polyclonal, ab18192, Abcam, WB: 1/1000), anti-DNA-PKcs (mouse monoclonal, ab1832-500, Abcam, WB: 1/200), anti-Vinculin (mouse monoclonal, V9131, Sigma-Aldrich, or mouse, monoclonal, sc73614, Santa Cruz, WB: 1/500), anti-Tubulin (mouse monoclonal, ab7291-100, Abcam, WB: 1/5000), horseradish peroxidase (HRP)-conjugated goat-anti-mouse (610-1319) or goat-anti-rabbit (611-1322) from Rockland (WB: 1/10000) and goat anti-mouse Fcγ from Jackson ImmunoResearch (WB: 1/10000), goat-anti-mouse Alexa 555 (A21424) or Alexa 488 (A32723), goat-anti-rabbit Alexa 555 (A21428) or Alexa 488 (A11008) from Invitrogen/Thermo Fisher Scientific (IF: 1/1000), IgG (Mouse, sc-2025 or rabbit sc-2027, Santa Cruz, for immunoprecipitation).

Validation

Antibodies were validated already in Eberle et al 2021 Front Oncol, Gole et al 2018 Leukemia and again in this work by the analysis of knockdown samples, expression of exogenous proteins or damage induction (Western blots in Source data, Extended Fig. 1, 4, 5, 7), omission of primary antibodies (PLA in Fig. 6d and Supplementary Fig. 9b).

Eukaryotic cell lines

Policy information about cell lines and Sex and Gender in Research

Cell line source(s)

K562 cells and HeLa cells were provided by Heinrich-Pette-Institute, Hamburg, Germany. HeLa cells for single-molecule tracking were purchased from Sigma-Aldrich/Merck. LentiX 293T cells were obtained from Clonetech Labratories Inc.

Authentication

K562 cells and HeLa cells as well as derived reporter cell lines were authenticated by Microsynth AG, Balgach, Switzerland, using STR profiling, except when directly purchased from Sigma-Aldrich/Merck.

Mycoplasma contamination

All cell lines were tested for mycoplasma contamination by PCR and confirmed to be negative.

Commonly misidentified lines
(See ICLAC register)

Authentication excluded misidentification.

Plants

Seed stocks

n/a

Novel plant genotypes

n/a

Authentication

n/a

Flow Cytometry

Plots

Confirm that:

☒ The axis labels state the marker and fluorochrome used (e.g. CD4-FITC).

☒ The axis scales are clearly visible. Include numbers along axes only for bottom left plot of group (a 'group' is an analysis of identical markers).

☒ All plots are contour plots with outliers or pseudocolor plots.

☒ A numerical value for number of cells or percentage (with statistics) is provided.

Methodology

Sample preparation	see Methods and references Eberle et al 2021 Front Oncol and Gole et al 2018 Leukemia
Instrument	FACSCalibur™ (BD Biosciences); CytoFLEX B3-R1-V0 flow cytometer with APD detectors and GFP-oD1 bandpass filter (Beckmann Coulter)
Software	BD Cell QuestTM Pro (BD Biosciences); CytExpert, Version 2.5.0.77 (Beckman Coulter)
Cell population abundance	see gating strategy
Gating strategy	Supplementary Figures 1b,c,d; 2b,c;

☒ Tick this box to confirm that a figure exemplifying the gating strategy is provided in the Supplementary Information.
