# Supplementary material for: Discovery of an Endonuclease G-inhibitory Ku80-peptide protecting against leukemogenic rearrangements at the MLL breakpoint cluster
Source: Nat Commun. 2026 Apr 17;17:3562. doi: 10.1038/s41467-026-72034-2 (PMC13086865; doi:10.1038/s41467-026-72034-2)
Supplement: Supplementary file 17 — Source Data [file 41467_2026_72034_MOESM17_ESM.zip › Source_Data_Uncropped_images.pdf]

Source Data: Uncropped Western Blots of Figure 1e and Figure 2a.

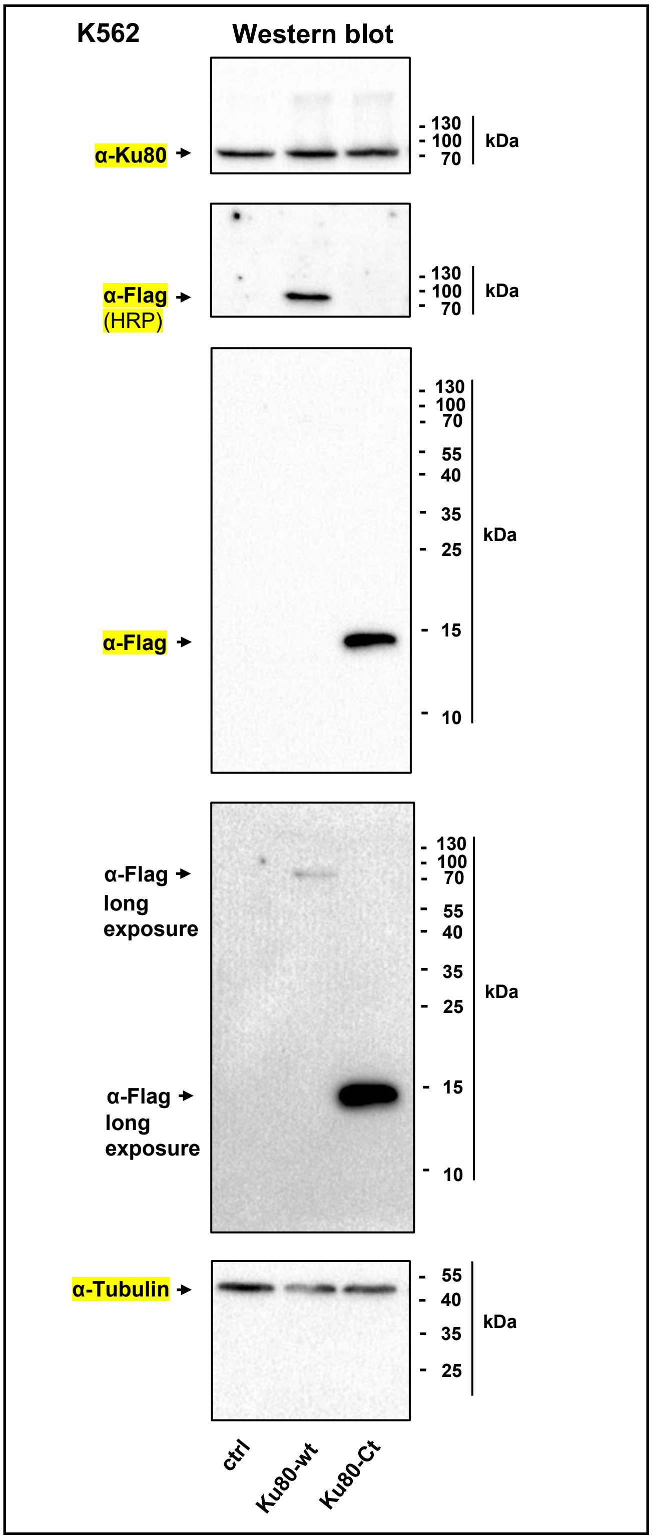

Source Data of Figure 1e: Uncropped Western Blot. Bands shown in Figure 1e are indicated by an arrow and highlighted in yellow. Long exposure of α-Flag immunoblot detects both tagged Ku80-wt and tagged Ku80-Ct. Primary antibodies used in Figure 1e and 2a are: α-Ku80 (Rabbit polyclonal, H-300, sc-9034, Santa Cruz), α-Flag M2 (mouse monoclonal, F1804, Sigma-Aldrich/Merck), α-Flag M2-Peroxidase (HRP, mouse monoclonal, A8592, Sigma-Aldrich/Merck), α-Tubulin (Mouse monoclonal, ab7291-100, Abcam).

Source Data of Figure 2a: Uncropped Western Blot. Bands shown in Figure 2a are indicated by an arrow and highlighted in yellow.

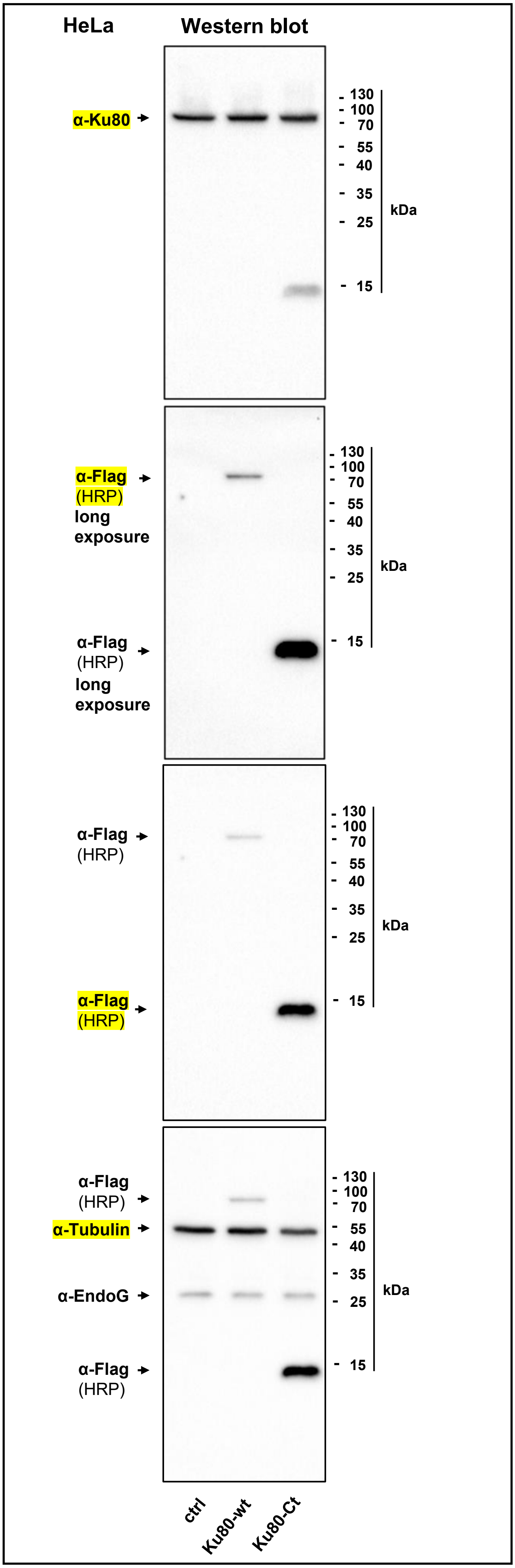

Source Data: Uncropped agarose gel images of Figure 2c, d.

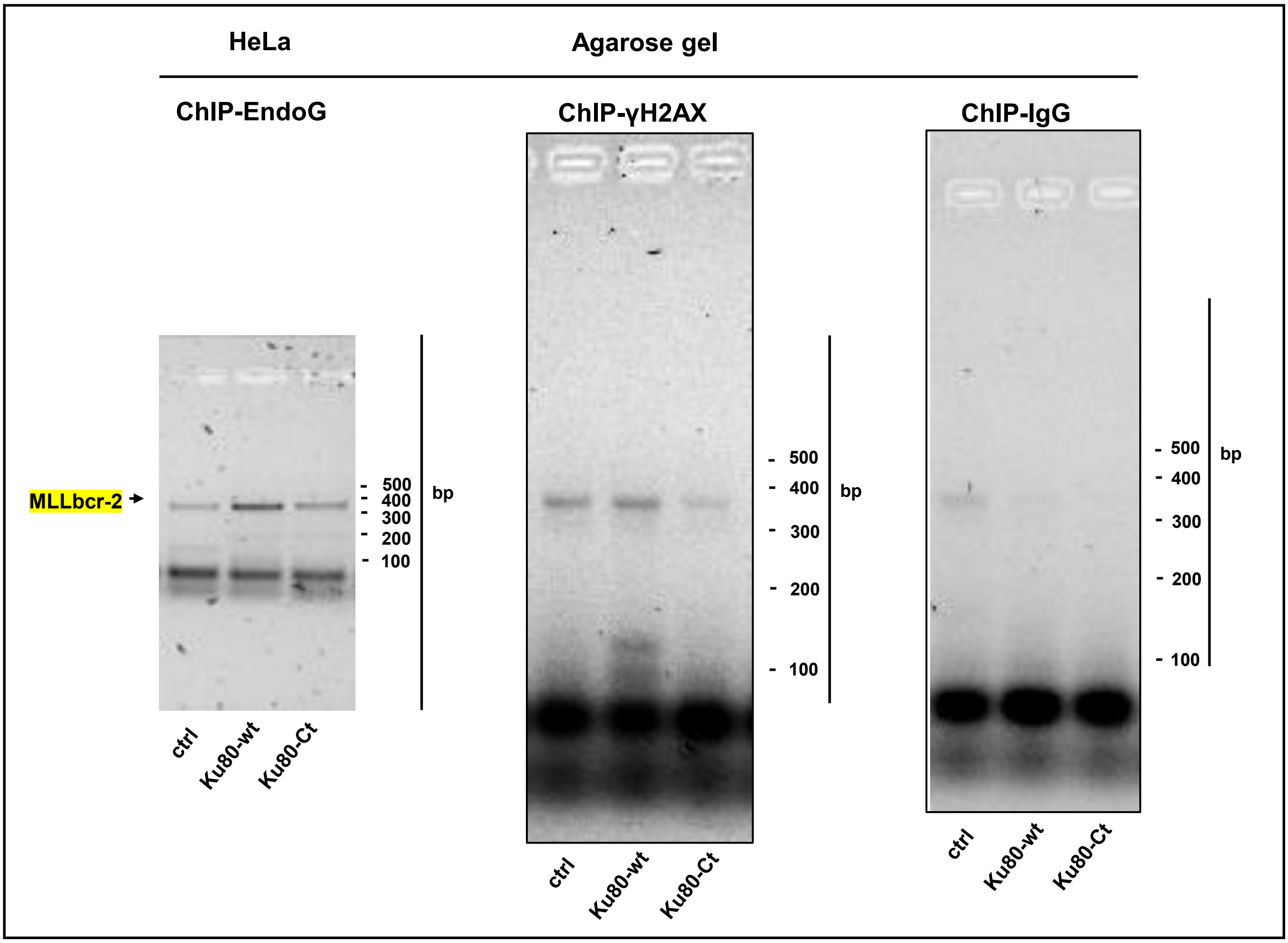

Source Data of Figure 2c: Uncropped agarose gel images. Bands shown in Figure 2c are indicated by an arrow and highlighted in yellow.

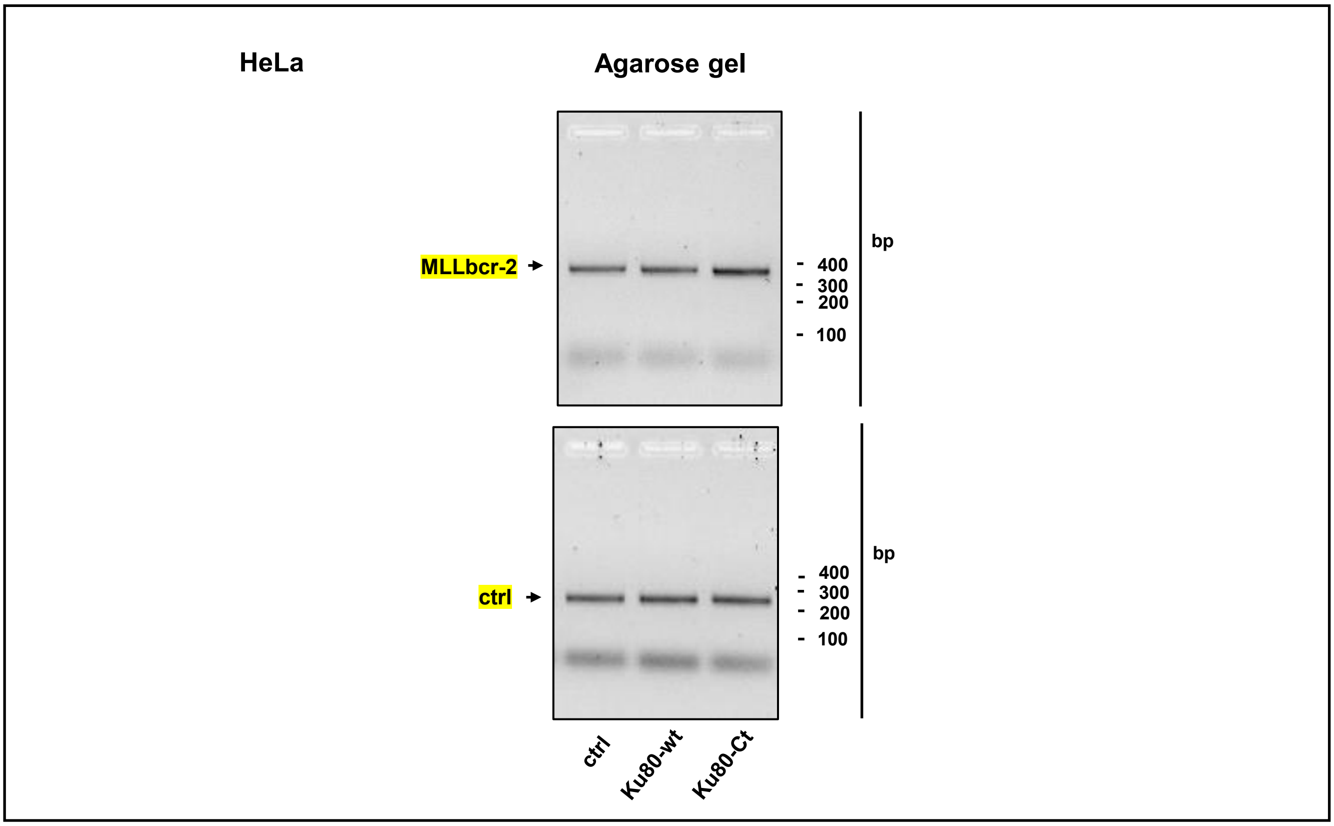

Source Data of Figure 2d: Uncropped agarose gel images. Bands shown in Figure 2d are indicated by an arrow and highlighted in yellow.

Source Data: Uncropped agarose gel images of Figure 5b and c.

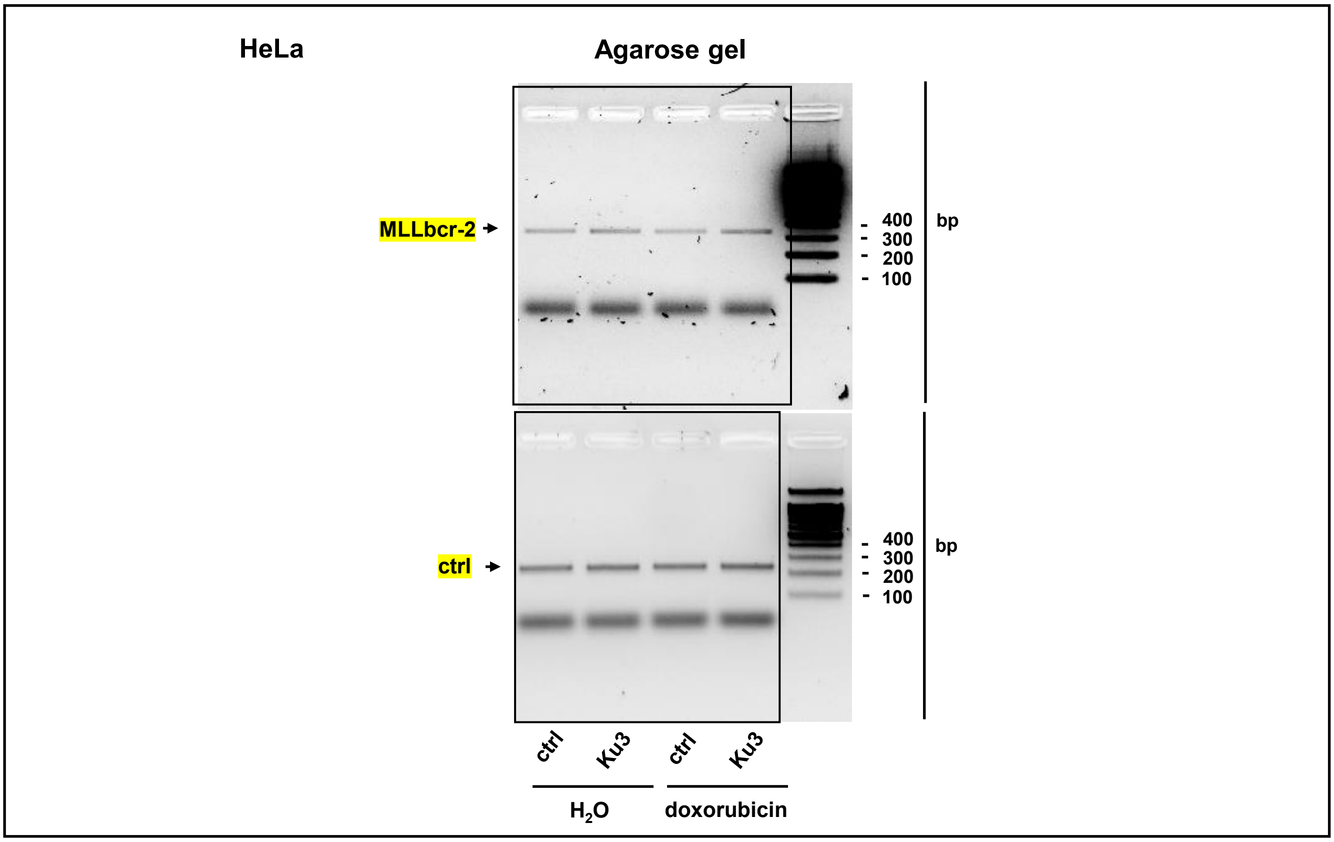

Source Data of Figure 5b: Uncropped agarose gel images. Bands shown in Figure 5b are indicated by an arrow and highlighted in yellow.

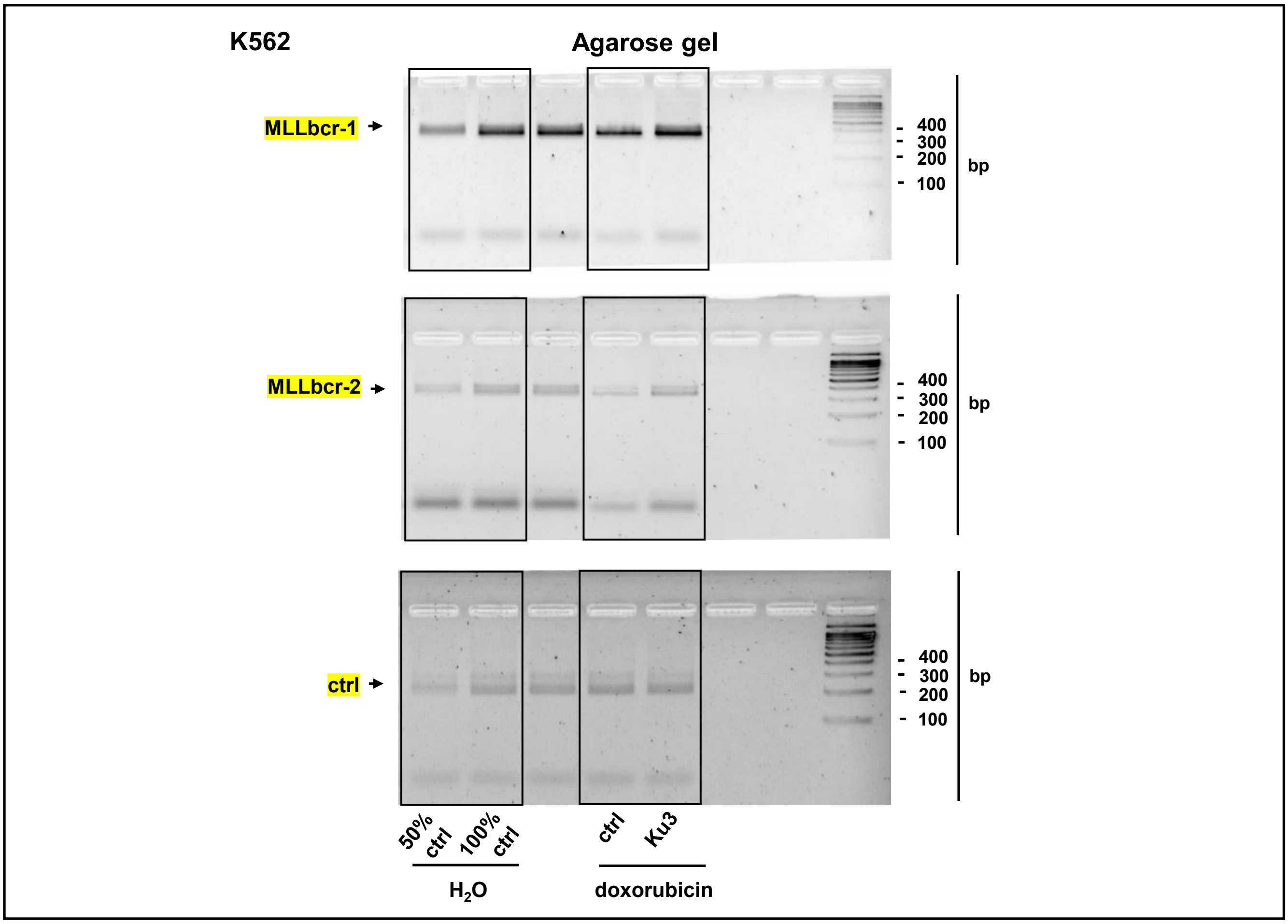

Source Data of Figure 5c: Uncropped agarose gel images. Bands shown in Figure 5c are indicated by an arrow and highlighted in yellow.

Source Data: Uncropped Western Blots of Supplementary Figure 1a.

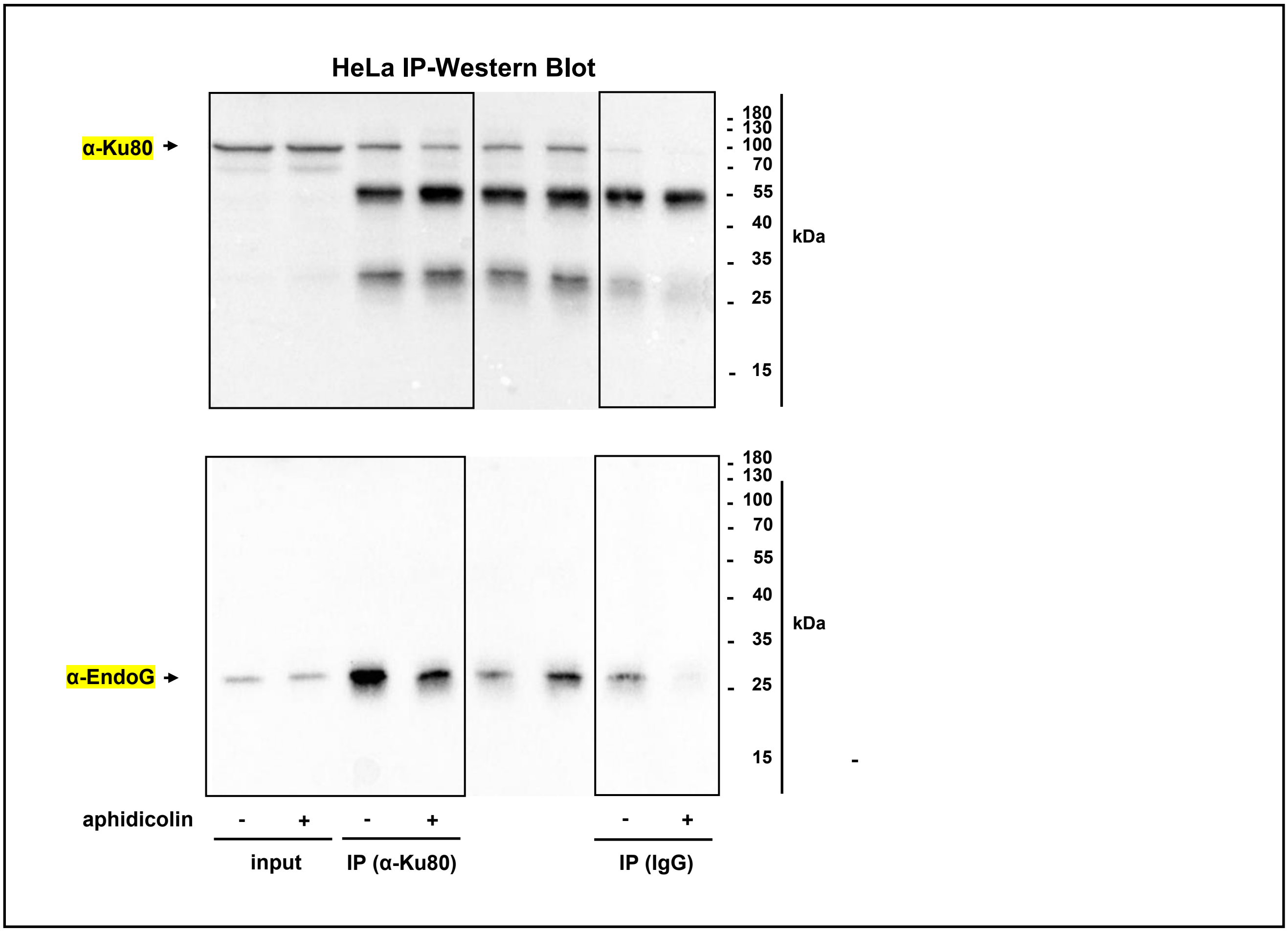

Source Data of Supplementary Figure 1a: Uncropped Western Blot. Bands shown in Supplementary Figure 1a are indicated by an arrow and highlighted in yellow. Primary antibodies used in Supplementary Figure 1a are: α-Ku80 (Rabbit polyclonal, H-300, sc-9034, Santa Cruz), α-EndoG (Mouse monoclonal, B-2, sc-365359, Santa Cruz).

Source Data: Uncropped Western Blots of Supplementary Figure 3b and 5b.

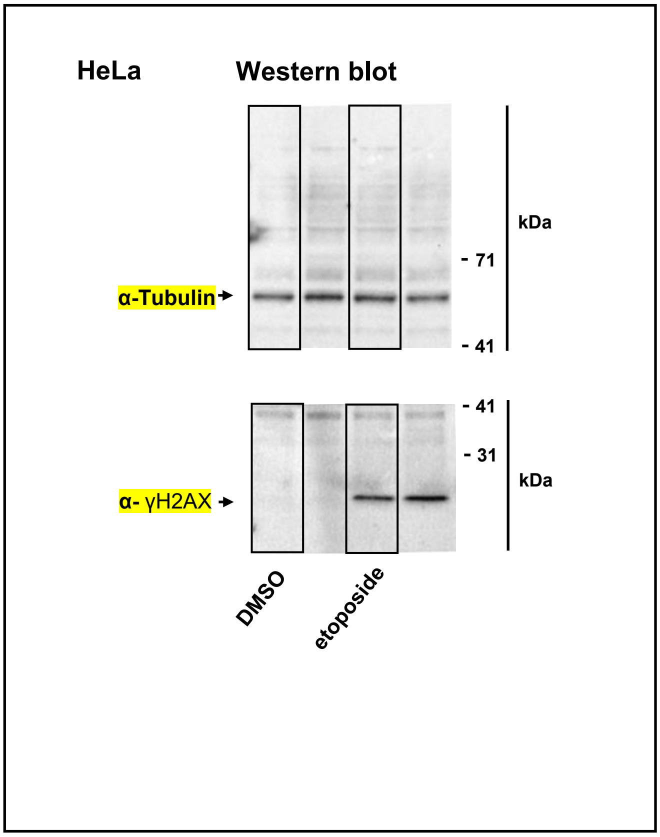

Source Data of Supplementary Figure 3b: Uncropped Western Blot. Bands shown in Supplementary Figure 3b are indicated by an arrow and highlighted in yellow. Primary antibodies used in Supplementary Figure 3b are: α-Tubulin (Mouse monoclonal, ab7291-100, Abcam), α-γH2AX Ser139 (Mouse, monoclonal, Clone JBW 301, 05-636, Merck Millipore).

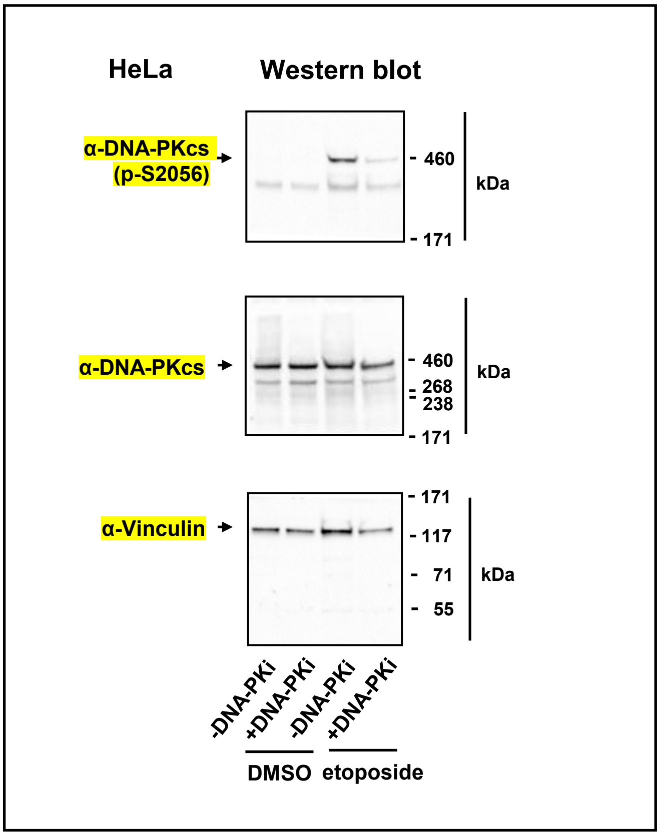

Source Data of Supplementary Figure 5b: Uncropped Western Blot. Bands shown in Supplementary Figure 5b are indicated by an arrow and highlighted in yellow. Primary antibodies used are: α-DNA-PKcs phospho S2056 (Rabbit, polyclonal, ab18192, Abcam), α-DNA-PKcs (mouse monoclonal, ab1832-500, Abcam), α-Vinculin (mouse monoclonal, sc73614, Santa Cruz).

Source Data: Uncropped agarose gel images of Supplementary Figure 5a.

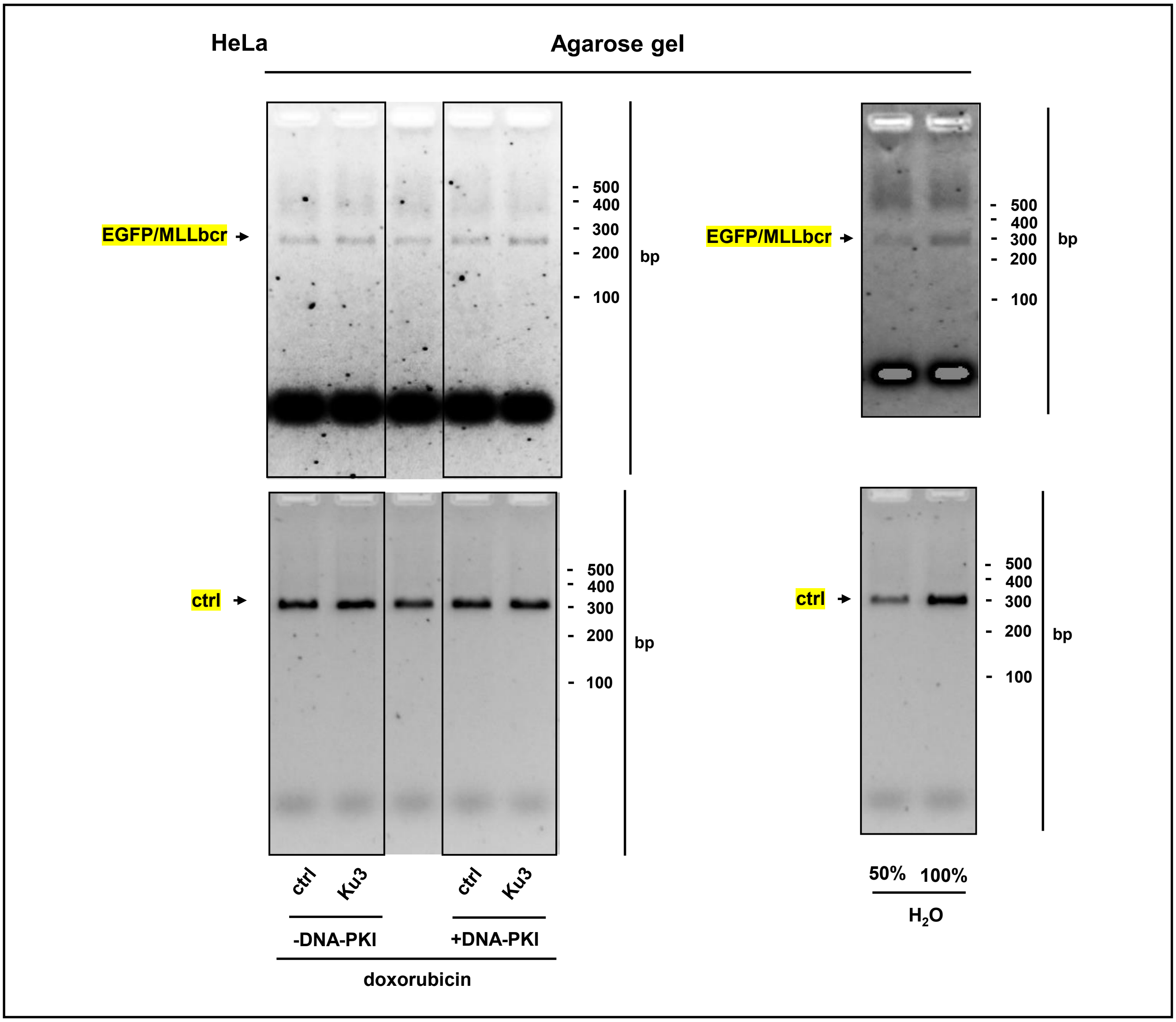

Source Data of Supplementary Figure 5a: Uncropped agarose gel images. Bands shown in Figure 5a are indicated by an arrow and highlighted in yellow.

Source Data: Uncropped Western Blot of Supplementary Figure 10a.

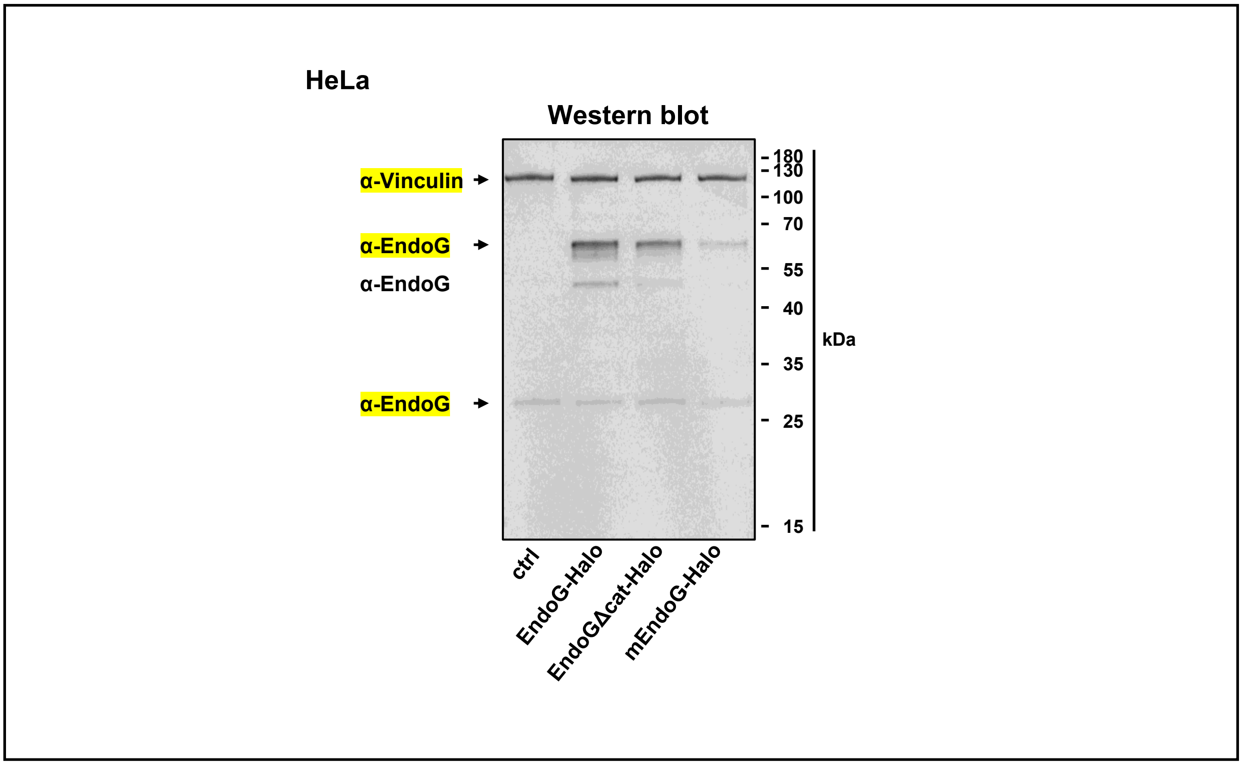

Source Data of Supplementary Figure 10a: Uncropped Western Blot. Bands shown in Supplementary Figure 10a are indicated by an arrow and highlighted in yellow. Primary antibodies used are: α-EndoG (Mouse monoclonal, sc-365359, Santa Cruz), α-Vinculin (mouse monoclonal, V9131, Sigma-Aldrich).
